# The Adipose Organ Is a Unitary Structure in Mice and Humans

**DOI:** 10.3390/biomedicines10092275

**Published:** 2022-09-14

**Authors:** A. Giordano, F. Cinti, R. Canese, G. Carpinelli, G. Colleluori, A. Di Vincenzo, G. Palombelli, I. Severi, M. Moretti, C. Redaelli, J. Partridge, M. C. Zingaretti, A. Agostini, F. Sternardi, A. Giovagnoni, S. Castorina, S. Cinti

**Affiliations:** 1 Department of Experimental and Clinical Medicine, Center for the Study of Obesity, Marche Polytechnic University, 60126 Ancona, Italy; 2 UOS Centro Malattie Endocrine e Metaboliche, UOC Endocrinologia e Diabetologia, Dipartimento di Scienze Mediche e Chirurgiche, Fondazione Policlinico Universitario A. Gemelli IRCCS, 00168 Roma, Italy; 3 Dipartimento di Medicina e Chirurgia Traslazionale, Università Cattolica del Sacro Cuore, 00168 Roma, Italy; 4 MRI Unit-Core Facilities, Istituto Superiore di Sanità, 00161 Roma, Italy; 5 Department of Public Health, Experimental and Forensic Medicine, University of Pavia, 27100 Pavia, Italy; 6 Anatomage Inc., Santa Clara, CA 95054, USA; 7 Department of Clinical, Special and Dental Sciences, Marche Polytechnic University, 60126 Ancona, Italy; 8 Department of Medical and Surgical Sciences and Advanced Technologies G.F. Ingrassia, University of Catania, 95121 Catania, Italy

**Keywords:** white adipocyte, brown adipocyte, beige adipocyte, mammary gland, organ, obesity, subcutaneous fat, visceral fat

## Abstract

Obesity is the fifth leading cause of death worldwide. In mice and humans with obesity, the adipose organ undergoes remarkable morpho-functional alterations. The comprehension of the adipose organ function and organization is of paramount importance to understand its pathology and formulate future therapeutic strategies. In the present study, we performed anatomical dissections, magnetic resonance imaging, computed axial tomography and histological and immunohistochemical assessments of humans and mouse adipose tissues. We demonstrate that most of the two types of adipose tissues (white, WAT and brown, BAT) form a large unitary structure fulfilling all the requirements necessary to be considered as a true organ in both species. A detailed analysis of the gross anatomy of mouse adipose organs in different pathophysiological conditions (normal, cold, pregnancy, obesity) shows that the organ consists of a unitary structure composed of different tissues: WAT, BAT, and glands (pregnancy). Data from autoptic dissection of 8 cadavers, 2 females and 6 males (Age: 37.5 ± 9.7, BMI: 23 ± 2.7 kg/m^2^) and from detailed digital dissection of 4 digitalized cadavers, 2 females and 2 males (Age: 39 ± 14.2 years, BMI: 22.8 ± 4.3 kg/m^2^) confirmed the mixed (WAT and BAT) composition and the unitary structure of the adipose organ also in humans. Considering the remarkable endocrine roles of WAT and BAT, the definition of the endocrine adipose organ would be even more appropriate in mice and humans.

## 1. Introduction

Triglycerides are highly energetic molecules used by mammals to survive during the intervals between meals and for non-shivering thermogenesis; they are stored in highly specialized cells called adipocytes. Adipocyte denomination derives from the characteristic abundance of lipids in the cytoplasm of this cell type. Two types of adipocytes have been described in mice and humans: white and brown [1]. White adipocytes show a unilocular lipid droplet occupying about 90% of their volume. Fatty acids released by this cell type allow survival during the intervals between meals. The second type of adipocyte has a multilocular lipid organization necessary to increase the lipid droplet surface, allowing the massive and rapid release of fatty acids to be used by mitochondria. Mitochondria contain an uncoupling protein called UCP1 responsible for energy dispersion in the form of heat from fatty acid oxidation [2]. Although the anatomy and physiology of white adipose tissue (WAT) and brown adipose tissue (BAT) are different, they cooperate to accomplish distinctive functions. During chronic cold exposure, WAT converts into BAT (browning) and during chronic excess of energy intake (obesity) BAT converts into WAT (whitening) [3,4,5]; this reversible conversion (mainly occurring through adipocyte transdifferentiation processes) may explain the close localization of WAT and BAT in mice and humans. For many decades anatomists described adipocytes as components of a specialized connective tissue, named adipose tissue, diffused among organs all over the body without any specific anatomic shape. Although the original definition of an adipose organ is due to Wells [6] that used this term to stress its metabolic importance, the very first anatomical demonstration that adipose tissues are contained in discrete and dissectible structures with specific anatomic shapes and size is quite recent [1]; these studies demonstrated that subcutaneous and visceral fat depots are well defined multiple structures, exhibiting two colors in animals maintained at room temperature: white and brown. The white parts of the organ are composed of WAT, while the brown ones by BAT [1].

The energy dispersion due to BAT activity and WAT browning can be exploited to curb murine obesity and type 2 diabetes (T2DM) [7,8,9,10,11,12] and several studies recently proved that browning exerts beneficial effects also in humans [13].

WAT and BAT are located in both subcutaneous and visceral depots, the last of which is here described as all fat depots in tight contact with viscera contained in the trunk. Several recent studies have outlined the different properties of visceral and subcutaneous fat mainly because visceral fat accumulation is associated with adverse metabolic outcomes [14].

Inspired by positron emission tomography (PET) images suggesting a continuity between visceral mediastinal fat and subcutaneous supraclavicular fat [15,16,17] we revisited the anatomy of the adipose organ of mice and humans to better define the relationships between the two compartments (subcutaneous and visceral). Considering the mixed composition of the organ (WAT and BAT) a detailed description of its anatomy including the precise localization of the two components will help understanding its physiology, pathology, and browning and whitening phenomena, holding potential therapeutic implications for obesity and associated disorders.

Our results demonstrate that the adipose tissues are organized to form a unitary dissectible structure in which most subcutaneous and visceral depots are in anatomical continuity in both mice and humans. Furthermore, we provide evidence that in mice BAT is mainly located in the “neckerchief” at the upper part of the body and in the periaortic sites, while in humans it is contained in periaortic sites where it is mixed with WAT. Finally, we show here the first 3D aspect of the human adipose organ derived from the study of serial sections on 3D planes of one adult human digitalized cadaver.

Considering the distinctive secretory abilities of subcutaneous and visceral fat as well as those of WAT and BAT and their interconvertible properties, the anatomical findings described here support a still unrecognized concept: the adipose organ represents the largest endocrine organ in mice and humans.

## 2. Materials and Methods

Given that our study was conducted on autoptic specimens and did not entail either an intervention, or the collection of the subject’s sensitive information, we have not obtained informed consent. In Italy, the evaluation of non-pharmacological observational studies is not governed by the same normative references provided for the evaluation of clinical trials and observational studies concerning drugs. Therefore, our Institutional Review Board does not require ethical approval for studies conducted on autoptic specimens and not collecting personal or sensitive data. Concerning animal experiments, mouse care was performed according to Council Directive 2010/63/UE and all procedures were approved by the Italian Ministry of Health (authorization no. 405/2018-PR).

### 2.1. Mouse Studies

A total of 22 mice, C57BL/6 (n. 20, 10 males and 10 females) and Sv129 (n. 2, one male and one female) were obtained from Charles River (Lecco, Italy); they were housed individually with a 12h light/dark cycle and with free access to food and water. Mice were euthanized with an overdose of anesthetic (ketamine) in combination with xylazine and immediately perfused transcardially with 4% paraformaldehyde in 0.1 M phosphate buffer, pH 7.4, for 3 min. The adipose organ was dissected under ophthalmic surgical microscopy (Zeiss OPI1, Carl Zeiss, Jena, Germany) to isolate all adipose tissues as a unitary organ from the rest of the body. After dissection, the adipose organ was disposed of on an ad hoc template and photographed by a Canon Camera (EOS 60D, EFS 18–55 mm), fixed at a stand for image capturing. Animals’ data and conditions are described in Supplementary Table S1.

#### 2.1.1. Light Microscopy and Immunohistochemistry

Samples of white and brown areas of the adipose organ were collected for histology and immunohistochemistry (UCP1) as previously described [18]. In brief: sections were reacted with 3% H_2_O_2_ (in dH_2_O; 5 min) to block endogenous peroxidase, rinsed with phosphate-buffered saline (PBS), and incubated in a 2% blocking solution (in PBS; 20 min). Sections were then incubated with the primary antibody against UCP1 (Abcam, Cat# ab10983; dilution 1:500) in PBS, overnight at 4 °C. After a thorough rinse in PBS, sections were incubated in a 1:200 *v*/*v* biotinylated secondary antibody solution (Vector Laboratories, Newark, NJ, USA) in PBS for 30 min. Histochemical reactions were performed using the Vectastain ABC kit (Vector Laboratories) and Sigma Fast 3,3-diaminobenzidine (Sigma-Aldrich, St Louis, MO, USA) as the substrate. Sections were finally counterstained with hematoxylin, dehydrated, and mounted in Eukitt (Fluka, St. Louis, MO, USA). Negative controls were included in each set of reactions by omitting the primary antibody. Selected fat sample fragments, showing subcutaneous-visceral connections (SVCs) were also processed for histology. Periaortic-mediastinal and parametrial-subcutaneous gluteal connections were paraffin-embedded, serial sectioned (every 10 μm) and H&E stained or used for immunohistochemistry staining. Furthermore, the whole bodies of two newborn mice (10 days) were fixed for 3 days in 4% paraformaldehyde after euthanasia by deep anesthesia. One was macroscopically sectioned in a medial sagittal line (head-tail) and the other in a transversal line (neck-thorax). The samples were dehydrated and infiltrated in paraffin. Sagittal sectioning was performed from median to outer sections, while transversal sectioning from neck to thorax. Serial sections were collected every 50 μm and stained with Hematoxylin and Eosin (H&E). The results derive from a 2D collage (Autostitch, Vancuver, Canada) of superimposable images, acquired at 2× magnification, that were used to further understand the topographic anatomy of adipose depots (Nikon Eclipse E800 light microscope equipped with a CCD camera, Nikon, Tokyo, Japan).

#### 2.1.2. Magnetic Resonance Imaging Method for the Study of the Adipose Organ of Mice In Vivo

Anatomy and fat composition were also studied in vivo by quantitative magnetic resonance imaging (MRI) and magnetic resonance spectroscopy (MRS). Ten weeks old C57BL/6 female mice were fed with a high-fat diet (HFD) for 18 weeks. In vivo assessment of fat composition in interscapular, peri-vesical, and inguinal fat was performed by quantitative 1H MRS after 18 weeks of HFD to better localize fat depots. MRI and MRS experiments were performed in mice using a Varian INOVA MRI/MRS system (Varian, Palo Alto, CA, USA) operating at 4.7 T with a transmitter volume RF coil actively decoupled from the receiver surface coil (RAPID Biomedical, Rimpar, Germany). Mice were anaesthetized by isoflurane 2.0% in O_2_, 1 L/min. Coronal (TR/TE = 600/18 ms, 4 transients, 23 slices, thickness = 0.8 mm, FOV 50 × 35 mm^2^, matrix 256 × 128) and axial multislice spin echo images (TR/TE = 700/18 ms, 4 transients, 29 slices, thickness = 0.8 mm, FOV 35 × 30 mm^2^, matrix 256 × 128) were acquired from the abdomen for quantitative evaluation of peri-vesical fat volume. Images were analyzed using the Varian VNMRJ 1.1D software. MRI/MRS methods for the study of the adipose organ of cold-acclimated mice: 10 weeks old female C57BL/6J mice were fed with HFD for 18 weeks. Mice return to be fed with chow diet (CD) simultaneously to cold-acclimation (CA) (15 days at 12 °C) and then to CD and standard temperature (22 °C) for one month. In vivo assessment of fat composition in interscapular, peri-vesical and inguinal fat was performed by quantitative 1H MRS after 18 weeks of HFD, at the end of CA and one month after the recovery. STEAM spectra with TR/TE/TM = 6000/9/10 ms have been used for water and lipid quantitative determination. LCModel was used for spectral fitting. The quantification of peri-vesical fat was assessed by MRI analyses. Histological analyses of subcutaneous and visceral fat were performed as described above.

### 2.2. Human Studies

Classical dissections from eight cadavers, 2 females and 6 males (Age: 37.5 ± 9.7 years, range: 27–56 years, BMI: 23.0 ± 2.7 kg/m^2^, range: 18.7–26.6 kg/m^2^) and sections from 4 digitalized cadavers (Age: 39 ± 14.2 years, range 26–59, BMI: 22.8 ± 4.3 kg/m^2^, range 20.3–27.8 kg/m^2^) by Anatomage (Anatomage Inc., Santa Clara, CA, USA) whose representative images and general characteristics are shown in Supplementary Figure S1 and in Supplementary Table S2. The data set of the two Caucasian digital cadavers was acquired within the Visible Human Project (VHP). After his death, the Caucasian man was frozen and serially sectioned at 1 mm intervals to obtain axial anatomical images [19], while the images of the female Caucasian cadaver were obtained at 0.33 mm intervals [20]. In the Visible Korean Human (VKH), the whole body of the Asian man was serially sectioned at 0.2 mm to obtain the data set of the axial images [21], while the female Asian body was acquired at 0.2 mm intervals from the head to the perineum and at 1 mm the lower limbs [22].

The Anatomage Table (AT) is the Virtual Dissection (Anatomage) Table projecting the whole body and the anatomical structures segmented in three dimensions for all these four cadavers built from the axial images of the VHP and the VKH. The bodies in the AT can be visualized as cross-sections in all planes, as well as 3-D reconstructions at any possible angle [23] allowing the user to virtually dissect a digital, life-size human cadaver and to navigate the 3-D cross-section anatomy of the four virtual human bodies [24]. In the AT, fantasy names were used for the Caucasian man and woman (Carl and Carla), and for the Asian man and woman (Victor and Vicky) as shown in Supplementary Figure S1.

#### 2.2.1. Digital Dissections of Virtual Cadavers

In the AT a total of 220 sections of Vicky were acquired (102 axial, 79 sagittal, 39 coronal). In all sections, fat depots were selected highlighting the border of their areas (Supplementary Figure S2) and verified by at least two of the most experienced authors. Selected areas were also studied at high magnification to confirm their fat nature (based on the color and the typical lobular organization). Based on the authors’ selection, Anatomage technicians extracted the whole-body adipose areas of both subcutaneous and visceral fat depots belonging to Vicky. For this purpose, the Anatomage software and the Zbrush (Pixologic Inc., Los Angeles, CA, USA) were used to refine the borders of the 3D model and to assess the inner volume of the fat model; this last step allowed us to visualize the whole adipose organ. One-hundred sixty-nine sections were created for Victor’s body (69 axial, 51 sagittal, 40 coronal). Fat was selected for all 169 sections. Ad hoc cross sections were acquired to better investigate the adipose tissue continuity regions in each body. Sagittal and transversal sections were analyzed (Vicky: 20–25, Victor: 20–25, Carl: 20–25, Carla: 20–25) to study the fat organization in the subcutaneous-visceral continuity areas. In addition, ad hoc cross sections were investigated for the cervical supraclavicular-mediastinal fat continuity 20–25 (Vicky: 4, Victor: 7, Carl: 4, Carla: 5). All sections were used to compare the four bodies.

#### 2.2.2. Classical Anatomical Dissections of Human Cadavers

Brown-like appearing areas of the adipose organ (selected based on the color observed in digitalized cadavers) and control areas (white appearing: periumbilical and gluteal) were sampled and studied by histology and immunohistochemistry in five cadavers (indicated by PV in Table S2) whose clinical data are shown in Supplementary Table S2. H&E-stained sections from each biopsy were studied. Selected samples (containing multilocular cells, Supplementary Table S4) were also evaluated by UCP1 immunohistochemistry as described above [18]. Tissue sections were observed with a Nikon Eclipse E800 light microscope. Morphometric analyses were performed as follows: the mean size of adipose cells was measured in all white adipose areas. From each slide stained with H&E five fields at 10× magnification were captured with a Nikon DXM 1220 camera and one hundred adipocytes were counted with the morphometric program ImageJ (RRID:SCR-003070). For the quantification of UCP1 positive areas, pictures of the whole UCP1-stained sections were taken at 4× magnification. The percentage of positive UCP1 cells in the total area was assessed by ImageJ.

#### 2.2.3. Magnetic Resonance Imaging in Humans

We selected 40 thorax MRI, 40 superior abdomen MRIs and 40 MRI of the pelvis exams, from a total of 120 subjects (49 females, 71 males, age: 63 ± 14 years, range: 44–89 years; BMI: 20–28 kg/m^2^). Samples were obtained from the Picture Archiving and Communication System (PACS) of the United Hospitals of Ancona, performed between the first of May and the 31st of December 2014. MRI images were evaluated to assess whether the adipose organ is a unitary structure in humans. Specifically, we looked for subcutaneous-visceral or visceral-visceral continuity between:

- periaortic fat and adipose tissue surrounding supraortic trunks in the thorax;
- periaortic and para-renal fat in the superior abdomen;
- periaortic and mesenteric fat in the superior abdomen;
- gluteal subcutaneous adipose tissue and visceral fat in pelvis.

The exclusion criteria were the presence of either pathological findings or post-surgical alterations of the nearby organs that could have altered the adipose MRI signal and/or the absence of MRI sequences performed with fat signal saturation. MRI exams were performed with a 1.5 T GE CVI/NVI tomograph (Software Excite HDXT 15.0, Waukesha, WI, USA), with a phased array coil 8 CH CARDIAC and with a 1.5 T PHILIPS ACHIEVA tomograph (Software 2.6.3.9, Eindhoven, Nederland), with a phased-array coil XL TORSO. All the exams were performed with T1SE and T2FSE sequences, with and without fat suppression, oriented in the three spatial planes. Fat suppression is an MRI technique that allows fat signal abolition, by introducing a radiofrequency band, that selectively saturates fat protons prior to acquiring data in standard sequences; this results in a black-dark gray signal allow to assess the presence and distribution of adipose tissue in the body [25] (Supplementary Figure S3).

#### 2.2.4. Computed Axial Tomography Scan in Humans

Forty chest and upper abdomen exams were selected from the Picture Archiving and Communications (PACS) archive of the Morgagni Polyclinic of Catania-Mediterranean Foundation “G.B. Morgagni, performed between 1 May and 31 December 2014. Data from 100 patients, 50 females and 50 males (age: 59.95 ± 5.17 years, BMI: 25.6 ± 3.0 kg/m^2^) were obtained.

We focused on the subcutaneous-visceral continuity (SVC) between mediastinal periaortic visceral fat and subcutaneous fat in the subclavian region through the fat enveloping collateral arteries of the aortic arch. Computed axial tomography (CAT) exams were performed with 64-slice volumetric acquisitions (SIEMENS TC SOMATON definition AS 64-Syngo acquisition software, Siemens, Bonn, Germany) with coronal thoraco-abdominal plane reconstruction.

## 3. Results

### 3.1. Mice

#### 3.1.1. Gross Anatomy

**Figure 1:**
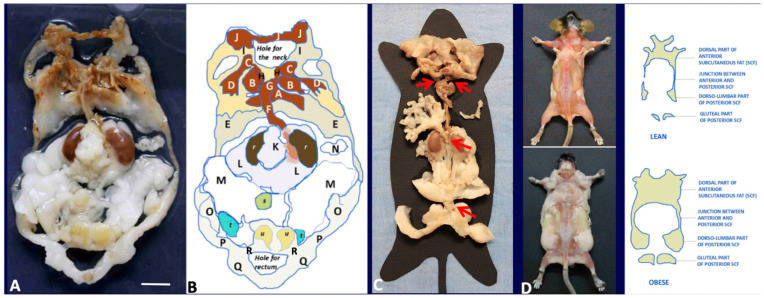
**Gross anatomy of mouse adipose organ in different experimental conditions.** (**A**): Anatomical preparation of the adipose organ as a unitary structure (adult, male C57BL/6 mouse, normoweight). Subcutaneous-visceral continuities (SVCs) are well visible and indicated in the diagram legend. Preputial glands and kidneys are left to facilitate anatomical orientation. White (WAT) and brown (BAT) adipose tissues are recognizable by their specific colors. (**B**): Legend: A: interscapular BAT (subcutaneous, SC), B: subscapular BAT (SC), C: supraclavicular BAT (SC), D: axillary BAT (SC), E: axillo-thoracic WAT (SC), F and G: periaortic mediastinal BAT (Visceral, V), H: SVC, I: cervical subcutaneous WAT (SC), J: cervical subcutaneous BAT (SC), K: mesenteric WAT (V), L: perirenal-retroperitoneal WAT-BAT (V), M: epididymal WAT (V), N: omental WAT (V), O: dorso-lumbar WAT (SC), P: inguinal WAT (SC), Q: gluteal WAT (SC). A + B + C + D + E + I + J: anterior subcutaneous region of adipose organ. O + P + Q: posterior subcutaneous region of the adipose organ. F + G + K + L + M + N: visceral region of the adipose organ. H and R: SVCs. r: kidney, s: urinary bladder, u: preputial glands. (**C**): Anatomical preparation of the adipose organ (adult, female C57BL/6 mouse, normoweight) as a unitary structure laying on a template. Red arrows point to SVCs (H and R in (**B**)) and mesenteric-abdominopelvic (inter-visceral) connection. (**D**): Dorsal view of adult C57BL6 female mice after skin removal. Anterior and posterior subcutaneous parts of the adipose organ are visible in both normoweight (upper panel) and obese animals. Bar: 8 mm in (**A**,**B**), 13 mm in (**C**) and 40 mm in (**D**).

In adult mice, 51% of the total fat weight is in the subcutaneous compartment (data not shown). The largest part of these depots is in the anterior and posterior part of the trunk, in the thigh connection with the upper and lower limbs (Figure 1).

**Figure 2:**
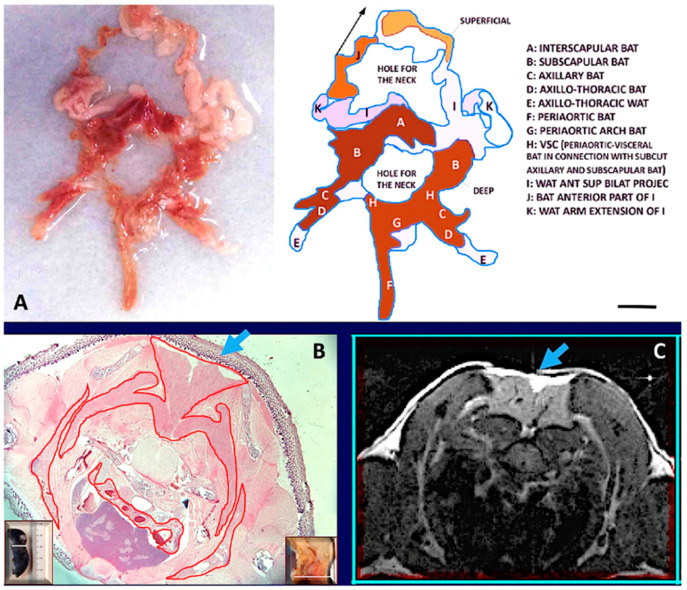
**The fat neckerchief of mouse Adipose Organ.** (**A**): Anatomical preparation of fat neckerchief displaying its continuity with peri-aortic mediastinal fat (adult C57BL/6 at room temperature). The superficial white fat of the neckerchief is separated and folded up as indicated by the arrow in the diagram. (**B**): Histology (newborn, C57BL/6) and axial T1-weighted MRI image (adult, C57BL/6) (in (**C**)) of the interscapular region, show the continuity between interscapular fat depot with the axillary fat, outlined by the red line surrounding brown fat in histology. Further evidence is shown by serial sections in histology and MRI (Supplementary Figure S7). The interscapular region (indicated by light blue arrows) also shows that brown (identified by a hypointense area) and white adipose tissue (hyperintense area) are contained in the same depot as also evident in anatomical preparations and histology. Insets in (**B**) show the plane of the section in the whole animal (left) and in its sagittal section (right). Bar: 10 mm in (**A**,**B**), and 1.5 mm in (**C**).

The anterior subcutaneous depot is quite complex, and its main mass occupies the dorsal region of the thorax in the interscapular area. From this central mass, several deep and superficial bilateral projections surround the thorax and reach the ventral region. The main interscapular mass has a pyramidal shape with the apex at the level of the second–3rd thoracic vertebra, corresponding to the emergence of a large vein (Sulzer), that is mainly devoted to drain blood from this depot. Deep bilateral projections extend under the scapulae and reach the fat in the anterior (ventral) supraclavicular-axillary region (Figure 2).

Other deep bilateral projections extend under nuchal muscles in the dorsal region of the neck (not shown). Superficial bilateral projections reach the anteroventral part of the body encircling the neck to join each other below the submandibular glands; these projections are in continuity with fat bands extending to the anterior legs (I and J in Figure 2A). A fat “neckerchief” thus surrounds the neck with white-brown superficial and brown deep parts. Posterior superficial bilateral projections encase the dorso-lateral part of the thorax ending ventrally in the axillae-thoracic depot (not shown).

The posterior subcutaneous depot is located at the base of the hind legs; its anatomy is simpler than that of the anterior subcutaneous depot; it is composed of a single tissue band beginning from the dorsum at the lumbar level (dorso-lumbar portion), extending ahead in the inguinal–crural region (inguinal portion), up to the pubic level into the gluteal region (gluteal portion). At the pubic level, this depot joins the contralateral one (Figure 1). The inguinal part extends above the suprapubic region where it encases the preputial glands in males (Figure 1). The gluteal part is joined to the pelvic visceral fat by small connecting bridges (Figure 1A,B: R in the scheme). The anterior and posterior subcutaneous depots are connected bilaterally by thin projections on the lateral part of the trunk (Figure 1D).

Visceral depots are contained in the trunk in tight connection with thoracic, abdominal, and pelvic organs. Most of the fat contained in the trunk surrounds the aorta and its main branches: brachiocephalic, left carotid, left subclavian, intercostal, renal, mesenteric. Of note, the peri-aortic mediastinal fat is in continuity with the peri-aortic abdominal fat through the aortic hiatus. In the female abdomen the peri-aortic fat surrounds kidneys and stands in continuity with mesenteric, retroperitoneal, periovarian, parametrial and peri-vesical fat. Most of this fat (excluding the mesenteric and retroperitoneal one) was previously described as abdominal-pelvic depot [1], thus here we extend this definition to include mesenteric and retroperitoneal fat. In the male abdomen the peri-aortic fat surrounds kidneys and is in continuity with the pelvic fat by the periureteral adipose tissue reaching peri-vesical fat; this latter is in continuity with the epididymal depot trough the fat surrounding the deferens ducts. All visceral fat depots contained into the trunk are in continuity with each other, except for omental fat, separated from the perirenal one due to the presence of the spleen. Collectively, the visceral fat mass consists of a unique structure composed by thoracic (periaortic) and abdominal-pelvic (peri-renal, retroperitoneal, mesenteric, periovarian, parametrial and peri-vesical or peri-ureteral and epididymal) parts (Figure 1).

**Figure 3:**
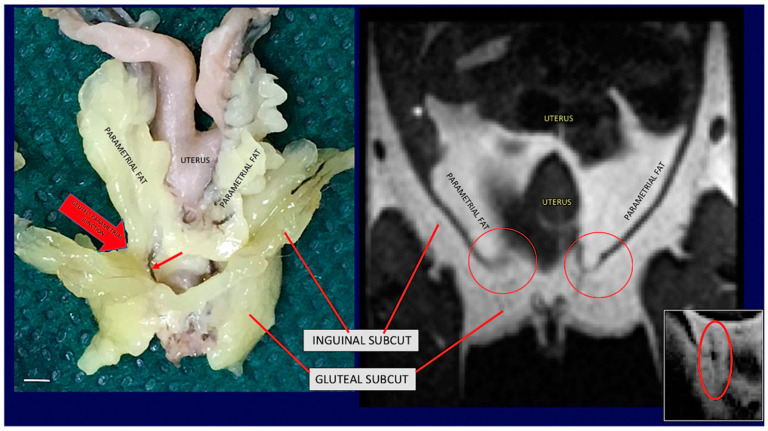
**Gross anatomy and MRI of subcuteous-visceral continuity in the lower part of mouse Adipose Organ.** (**Left**): Anatomical preparation of an adult C57BL/6 mouse showing subcutaneous-visceral continuity between parametrial and gluteal fat. (**Right**): coronal T1-weighted MRI of the same areas shown in the left panel of the same animal in vivo. Anatomical continuity is indicated in the visceral (parametrial) and subcutaneous (inguinal) fat by arrow and red circles. A small red arrow on the left panel shows a vessel in the junction. A similar structure was also detected by MRI (red circled area in small, squared panel on the right). Bar 4.5 mm in both panels.

The unitary visceral fat mass Is in continuity with the subcutaneous depots at two sites: at the root of the neck and at the lower opening of the pelvis (Figure 1C red arrows and Figure 3).

At the root of the neck fat surrounding the aorta arch and its branches provides the continuity between the mediastinal visceral fat with the subcutaneous supraclavicular-axillary fat (i.e., fat surrounding subclavian and axillary vascular-nerve bundles). At the lower opening of the pelvis, peri-vesical fat is joined to the gluteal part of the posterior subcutaneous depot by an evident bridge of fat surrounding the lower part of the rectum (Figure 1 and Figure 3). Further evidence of the unitary organization of mouse adipose depots is provided by the anatomical dissection of the adipose organ as a whole structure (Figure 1). The reported description of the gross anatomy of the adipose organ is valid for all dissected C57BL/6 and SV129 mouse strains.

#### 3.1.2. Histology

WAT and BAT are believed to form independent anatomic structures. However, based on the findings described above, WAT and BAT are contained in different areas of the same organ, hence form a single structure. White was the prevalent color of the organ, but the brown color was present in several areas of the anterior subcutaneous depot (interscapular, subscapular, deep cervical, and sub-clavicular-axillary) and in several visceral areas (mediastinal, periaortic, perirenal, mainly in the inter-renal area). In young mice, the anterior mediastinal-supraclavicular-axillary connection is composed by brown fat based on our histological data (Supplementary Figure S4). Histology showed that all the brown areas were composed mainly of multilocular adipocytes, and that all the white areas were composed by unilocular adipocytes. Immunohistochemistry showed that multilocular adipocytes were mainly UCP1 immunoreactive, whereas unilocular adipocytes were UCP1 negative. Interestingly, areas of transition between the white and brown regions contained all different intermediate cellular phenotypes between white and brown adipocytes, exhibiting variable UCP1 immunoreactivity (Supplementary Figure S5). In addition, some white areas (mainly inguinal and abdominal-pelvic) showed UCP1 immunoreactive multilocular cells dispersed among white adipocytes. The SVC between gluteal (subcutaneous) and pelvic (visceral) fat is formed by white fat. In female mice the presence of mammary ducts was evident only in the gluteal part of this gluteal-pelvic connection (Supplementary Figure S6), in line with the well-known notion that the gluteal fat area contains the fifth mammary gland in females [26]. Of note, vascular structures connecting the gluteal with the pelvic fat were also evident by MRI (Supplementary Figure S3).

#### 3.1.3. Magnetic Resonance Imaging

Total body T1 weighted MRI analyses of adult mice of both sexes confirmed the above-described gross anatomy of the adipose organ in vivo. Specifically, the fat neckerchief connecting the anterior supraclavicular fat with the dorsal part of the anterior subcutaneous fat was observed (Figure 2). Furthermore, the upper SVC (connecting the subcutaneous supraclavicular fat area with the periaortic mediastinal visceral fat area) (Supplementary Figures S4 and S7) and the lower SVC (connecting the pelvic fat area with the gluteal fat area) were also documented (Figure 2 and Figure 3 and Supplementary Figure S7).

#### 3.1.4. Magnetic Resonance Imaging Following Cold Exposure

After 7–10 days of CA (6 °C), in adult (8 weeks old) and old (24 weeks old) mice (C57BL/6 and SV129) of both genders, the color of the adipose organ turned brown (browning) in several areas (Supplementary Figures S8 and S9). The brownest parts of the adipose organ were confined to the anterior subcutaneous area including the interscapular, subscapular, deep, and superficial cervical and axillary depots, all together forming the “neckerchief” shown in (Figure 2). Furthermore, all areas surrounding the aorta and its main branches were also visibly brown (Figure 2 and Supplementary Figure S4). Interestingly, the percentage of whole visceral fat increased suggesting proliferation of BAT and/or shrinkage of subcutaneous fat (data not shown). Of note, all the above-described SVCs were also found following CA (Supplementary Figures S8 and S9). MRI analyses showed that, despite the similar body weight (ranging between 24.5 and 25.5 g) maintained during the entire period, the water over fat ratio (water/lipid signal at 0.9 ppm, an indirect index of BAT), changed after CA, as it increased in subcutaneous inguinal area (Supplementary Figure S10). In peri-vesical fat, this ratio is usually low (due to the massive presence of WAT) also in normofed animals. In the condition of CA, we observed an increase in water/lipid signal at 0.9 ppm in the peri-vesical fat as well; these results were confirmed by histology of peri-vesical region (not shown). Following CA, we also observed an increase in the water/lipid ratio in the interscapular region reflecting an increase in the BAT fraction (Supplementary Figure S11).

#### 3.1.5. Gross Anatomy in Obesity

The adipose organ of obese mice confirmed the gross anatomy observed in normoweight animals (Supplementary Figure S8B) although its size and weight were higher (~three times, data not shown). In these animals the SVC at the pelvic-gluteal level was more evident, especially in females (Supplementary Figure S8B). The brown parts were reduced and mainly restricted to the periaortic fat and to the deep parts of the depots forming the “neckerchief” compared to normoweight. In these areas immunohistochemistry showed a small amount of multilocular adipocytes weakly immunoreactive for UCP1 (data not shown). Consistently, the MRI/MRSA data showed a reduction of the water/lipid signal in the subcutaneous inguinal area (Supplementary Figure S10).

#### 3.1.6. Gross Anatomy in Lactation

Data from our laboratory suggest that during pregnancy and lactation suggest most subcutaneous fat converts into epithelial glandular cells forming the alveolar part of mammary glands, even though the matter is still quite controversial [27]. As expected, all visceral depots were not affected by this conversion, and periaortic (including SVC), perirenal, mesenteric, parametrial and peri-vesical fat was present in small quantity (Supplementary Figure S12). Interestingly, the deep part of interscapular, subscapular and axillary fat were brown and unaffected by any adipo-glandular conversion or glandular development (data not shown).

### 3.2. Humans

#### 3.2.1. Gross Anatomy

**Figure 4:**
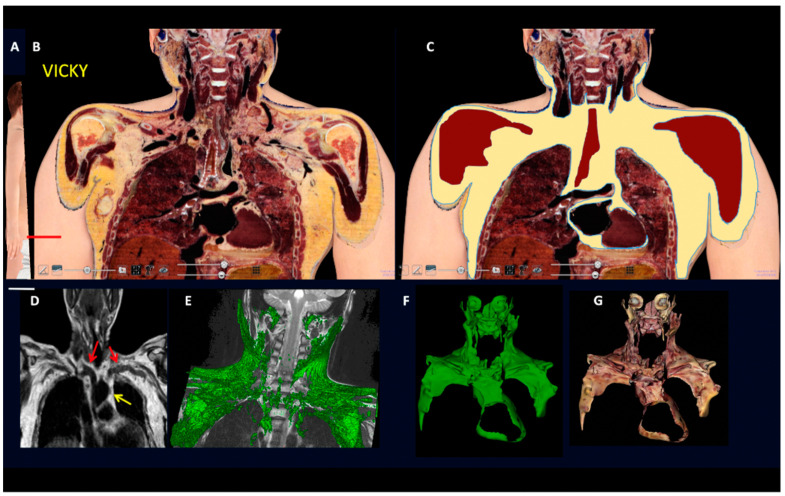
**Gross anatomy and MRI of subcutaneous-visceral continuity in human Adipose Organ.** (**A**): Oblique frontal section of a digital cadaver (Vicky). (**B**): Supraclavicular-mediastinal continuity of the section shown in (**A**). (**C**): The same section shown in (**B**), with the fat area highlighted in yellow. (**D**): Routine MRI in an adult male. Red arrows indicate subcutaneous-visceral fat connection (SVC) and the yellow arrow indicates the mediastinal fat. (**E**): 3D rendering (green) of the SVC in a 10-year-old boy. (**F**,**G**): 3D reconstruction of the fat present in the supraclavicular-mediastinal continuity shown in (**B**,**C**). Fat area is highlighted in three planes (axial, coronal, and sagittal); in F fat is in green for comparison with (**E**); in (**G**) fat is shown in its original colors. Bar: 60 mm in (**B**,**C**), 45 mm in (**F**,**G**).

**Figure 5:**
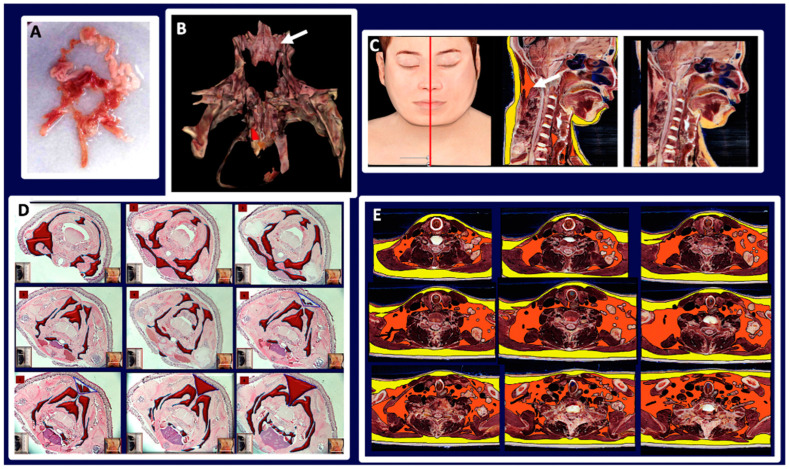
**Comparative data between mouse and human fat neckerchief.** (**A**): Human fat neckerchief. (**B**): 3D reconstruction of the fat areas obtained through the entire set of Vicky’s cervical sections. Dorsal view, the deep neck brown fat mass is indicated by the arrow. (**C**): Vicky median sagittal section (left panel) in which the subcutaneous fat is highlighted in yellow and the brown fat in orange (central panel). The arrow indicates the deep neck brown fat also shown in (**B**). The right panel shows the original section with no highlights. (**D**,**E**) show a comparison between the brown fat forming the neckerchief in a mouse and in Vicky serial sections (original sections are shown in Supplementary Figures S7 and S15). Bars: 15 mm in (**A**), 60 mm in (**B**), 14 cm in (**C**), 15 mm in (**D**), 6 cm in (**E**).

**Figure 6:**
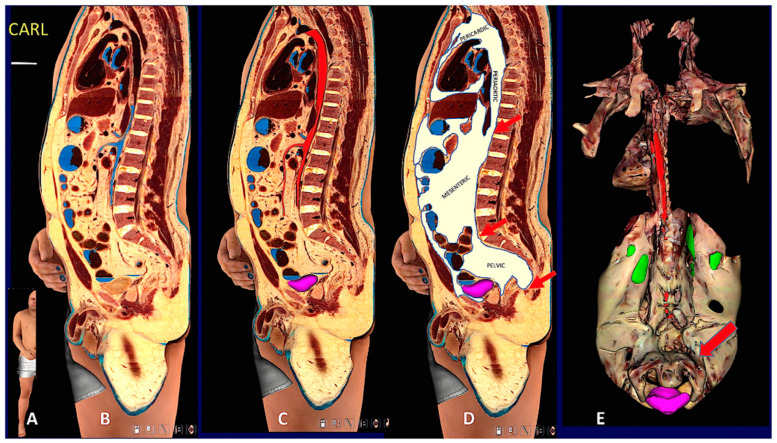
**Digital cadaver section and 3D reconstruction showing upper and lower subcutaneous visceral continuity in the Adipose Organ.** (**A**): Sagittal oblique section of the digital cadaver, Carl. (**B**–**D**): Mediastinal-abdomino-pelvic and gluteal subcutaneous-visceral connection (SVC). (**B**): original section, (**C**): aorta and upper mesenteric artery in red, (**D**): fat highlighted in white. Arrows in (**D**) point to mediastinic-abdominal (upper), abdomino-pelvic (middle) and gluteal-pelvic (lower) connections. Gluteal-pelvic SVC is also shown in Supplementary Figure S16. Panel (**E**) shows a 3D reconstruction of the whole visceral fat. Dorsal view in which the continuity between retroperitoneal and pelvic fat surrounding the urinary bladder is more evident, indicated by the arrow. Green: kidneys (parts of dorsal surface not covered by retroperitoneal fat), violet: urinary bladder. Arrows point to periaortic fat in the mediastinic-abdominal connection. See also Suppl movie. Bar: in (**B**–**D**) 60 mm, in (**E**) 120 mm.

**Figure 7:**
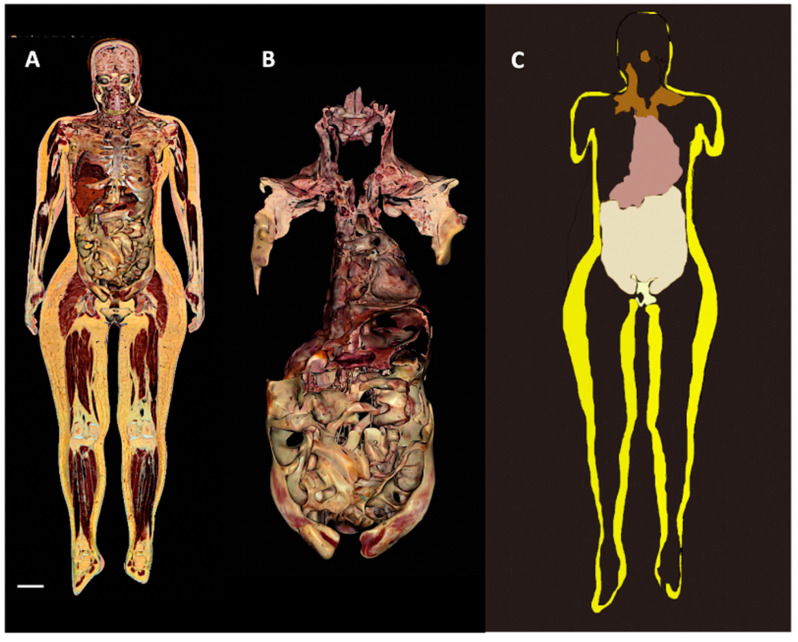
**2D and 3D reconstructions of human Adipose Organ.** Digital cadaver Vicky. (**A**): Representative images showing the whole adipose organ in a coronal section, (**B**): the 3D reconstruction of isolated visceral fat in connection with the supraclavicular-axillary part of the adipose organ, and (**C**): the visceral (brown, beige, and pale white) and subcutaneous (yellow) parts of the adipose organ (still image from S Movie 1). Bar: 65 mm in (**A**), 35 mm in (**B**) and 75 mm in (**C**).

In line with the largely accepted notion that subcutaneous fat forms a continuum layer under the skin of the whole body in humans [28], our analyses and a 3D reconstruction of the whole subcutaneous part of the adipose organ properly reproduced the shape of the intact body (Supplementary Figure S13). Notably, an evident difference between human and mice subcutaneous fat consisted of the lack of an interscapular depot in the former, where only a small pyramidal-shaped thickening of the deeper layer of the subcutaneous fat was found (data not shown). All inter-visceral depots communications described for mice were found also in humans (Figure 4, Figure 5, Figure 6 and Figure 7 and Supplementary Figures S14–S18 and S20, S21).

Although the SVCs was observed in some PET analyses [15,16,17], they have never been accurately described. We thus assessed whether the SVCs that we observed in mice were also present in humans. Specifically, we assessed whether SVCs were visible in classic and digital dissections of cadavers and in living adult subjects by MRI and CT imaging techniques, gold standard methods for the study of body composition [29,30].

All classic and digitalized anatomical dissections of the 12 adult cadavers showed SVCs corresponding to those described in mice (Figure 4, Figure 5 and Figure 6) and displayed very similar aspects (Supplementary Figures S14 and S15). Interestingly, most of the SVCs in the cervical-mediastinal area had a brown color like the one described in the neckerchief fat of mice. Another region displaying brown features was localized in the deep cervical area; this brown aspect was particularly evident in both young and normoweight digital cadavers examined (Vicky and Victor) and was also confirmed in classic dissections. We thus studied autoptic specimens from cadavers considering that the anthropometric data were similar to those of the two digital cadavers (Vicky and Victor, see Supplementary Table S2). Abdominal-pelvic (inter-visceral) and pelvic-gluteal (subcutaneous-visceral) continuities were easily visible in both coronal and sagittal sections, as well as in the 3D reconstruction obtained by the serial sections of the three planes (Vicky, Supplementary Figures S16 and S17). The 3D reconstruction of the whole adipose organ of Vicky, obtained by the serial sections on three planes (axial, sagittal, and coronal) is presented in the Supplementary Movie S1. An image from the movie, summarizing the main aspects of the whole adipose organ of an adult human, is shown in Figure 7. The anatomical preparation of the upper SVC (mediastinal-supraclavicular) from classic anatomical dissections of a human cadaver (case 1 PV, Supplementary Table S2) is presented in Supplementary Figure S18. Visceral and subcutaneous fat volumes of the digital cadaver Vicky are described in Supplementary Table S3.

#### 3.2.2. Histology

Histology and immunohistochemistry, performed on the eight areas indicated in the Supplementary Table S4 from the five cadavers (PV, Supplementary Table S2), showed the presence of a mixture of unilocular (white-like) and multilocular (brown-like) adipocytes in most of the sampled areas (including peri-carotid, peri-subclavian, peri-aortic, axillary, and inter-renal). Periumbilical and gluteal areas were used as controls (white fat) (Supplementary Table S4). Of note, we observed UCP1 positive multilocular adipocytes (brown adipocytes) and many UCP1 positive unilocular and paucilocular cells, like those recently described in the perirenal adipose tissue of a Siberian patients [31] (Supplementary Figure S19). Quantitative assessment of UCP1 positive adipocytes revealed that most of the brownish areas in digital cadavers contained UCP1 immunoreactive cells (multilocular and paucilocular) in the five cadavers. Inter-renal fat was the depot with the highest number of UCP1 immunoreactive adipocytes (Supplementary Table S4). Furthermore, the sampled areas lacking evidence of brown adipocytes were composed by very small unilocular adipocytes (2485.6 µm^2^ ± 1241.7 vs. 5058 µm^2^ ± 1074.5 in the abdominal and gluteal subcutaneous fat (*p* = 0.03).

#### 3.2.3. Magnetic Resonance Imaging

MRI showed clear SVCs in the thorax, superior abdomen, and pelvis from all adult patients of both genders. Furthermore, all truncal inter-visceral communications were also identified (Supplementary Figure S20).

#### 3.2.4. Computed Axial Tomography

In all 40 selected CAT scans we confirmed the presence of the SVCs described by AT and MRI analyses. Specifically, in the sub clavicular area, around the aortic arch collaterals, we found a “hypodense” tissue (HU-100) consistent with adipose tissue in continuity with the supraclavicular space, bilaterally along the subclavian and common carotid vessels (Supplementary Figure S21).

## 4. Discussion

Obesity and associated disorders have spread as a pandemic and represent the fifth leading cause of death worldwide [32,33,34]. For this reason, therapeutic strategies are urgently needed [35]. Obesity is characterized by the aberrant expansion of the adipose tissues due to a chronically positive energy balance [32]. Thorough knowledge of adipose tissues organization, distribution and cellular composition is necessary to exploit cytological data for therapeutic purposes. The anatomical data presented here strongly support the idea that the largest amount of fat in mammals is organized to form a large unitary organ, in agreement with several previously published analyses in mice and humans in vivo; this finding implies at least two new concepts: 1-WAT and BAT are contained together in the same organ, 2-visceral and subcutaneous fat are organized as a continuum in the same organ. Traditionally, BAT has been described as an anatomical entity separated from WAT. In striking contrast, here we demonstrate that in both subcutaneous and visceral compartments, WAT and BAT are contained in the same depots as different entities but in continuity between each other. Of note, all the peripheral areas, where each tissue was enriched in intermediate forms between white and brown adipocytes, strongly resemble those described in the process of browning or whitening.

The comprehension of the anatomical organization and composition of the adipose organ is of critical relevance. In the condition of chronic positive energy balance, adipocytes display a different susceptibility to hypertrophy and hyperplasia, which depends not only on age and gender, but also on their location within the adipose organ [36,37]. For example, obesity severity, hence, the risk of development of metabolic complications, is higher in the condition of visceral compared to subcutaneous obesity [14]. Indeed, compared to subcutaneous adipocytes, hypertrophic visceral adipocytes display a lower *critical death size,* above which cells die and trigger macrophage infiltration [38,39]. Macrophage infiltration and inflammation are in turn associated with the development of insulin resistance and T2DM [40,41], the most frequent conditions associated with obesity [42,43].

An additional modification of the adipose organ documented in condition of obesity involves BAT depots, whose mass reduces and phenotypical appearance shifts resembling white cells [5,44]. Importantly, the study of BAT organization and activity is of great clinical significance, as its activation can increase total energy expenditure [7]. The re-discovery that metabolically active BAT is present in adult humans [45,46,47] renewed the hope for the identification of pharmacologic stimuli able to activate BAT to curb obesity and related disorders [8,9,10,11,12,48,49,50]. In experimental animals, BAT activation involves fat burning and ameliorates both diet-induced and genetic obesity [10]. BAT activation results in an increased expression of UCP1, brown adipocyte proliferation, and in a phenotypic transition of white adipocytes belonging to specific depots into brown-like cells [1]. During this last conversion, several intermediate stages between the initial hypertrophic white adipocyte and the final small brown-like cells are documented and include small white-like adipocytes rich in mitochondria which were named paucilocular adipocytes by our group [3]. Paucilocular adipocytes can be immunoreactive for UCP1 [3,31] and their activation through beta3 adrenergic agonists could have a therapeutic efficacy even when only a partial conversion of white unilocular adipocytes into brown-like paucilocular adipocytes is reached.

The mixed composition of the adipose organ requires a teleological explanation, and we proposed the following: the ability of adipocytes to convert reciprocally under physiologic stimuli allows them to respond to specific environmental requirements. Specifically, chronic cold induces browning, whereas chronic positive energy balance induces whitening [1]. Additional, and not-mutually exclusive explanations of such topographical organization are the following: 1. peripheral WAT works as insulator avoiding the dispersion of the heat produced by BAT activation; 2. white adipocytes close to and intermingled with brown adipocytes potentially fuel energy substrates and signaling molecules to adjacent thermogenic cells to sustain and regulate thermogenesis. The macroscopical and microscopical analysis of the adipose organ of cold exposed and obese mice, together with in vivo data from mice by MRS, agree with the browning and whitening phenomena. Of note, the most persistent (with age) brown part of the organ is in the deep part of the fat neckerchief and in the region tightly connected with aorta and its main branches; this offers an easy teleological explanation: heat produced by BAT can rapidly be transported everywhere in the body by bloodstream through the aorta and Sulzer vein. In humans most of the collar-neckerchief is absent and PET analyses of cold-exposed subjects show a BAT signal at the periaortic location [15]. The fat able to undergo the highest conversion in humans is thus the peri-aortic visceral fat. In this context it should be outlined that there is a consensus that subcutaneous fat is more prone than visceral fat to browning [51]; this statement is, however mainly based on the unresponsiveness of the most studied visceral depot in mice: the epididymal fat. However, based on our data the visceral periaortic fat (including fat surrounding the main aortic branches) is highly prone to browning. Some Authors tend to restrict the term visceral to the abdominal fat whose venous blood is drained by the portal vein, mainly because of the well-known specific morbidity associated to non- alcoholic steatohepatitis and abdominal obesity. We think that the presented anatomical continuity among most of the intra-truncal fat depots play against any restriction of visceral denomination. Most fat surrounding the aorta and its main branches in young or cold exposed mice is BAT whose whitening results into small unilocular cells like those found in visceral fat depots [5]. The well-recognized difference between subcutaneous and visceral fat is that this last is formed by smaller unilocular adipocytes provided with a denser vasculature and innervation [1]; it is hence possible that most of the visceral fat derives from the whitening of BAT. In this case, the browning of visceral fat could be an important therapeutic target for obesity and related disorders as whitened BAT would be more prone to convert back to the brown phenotype. Furthermore, given the lower critical death size of visceral adipocytes, their browning would have critical clinical implications as it would reduce their death and inflammation, hence insulin resistance and dyslipidemia. In this context it is interesting that Cypess et al. showed that a single oral dose of the last generation beta3 adrenergic receptor agonist could induce a massive activation of periaortic BAT in young and normoweight volunteers [15]. In line with these data, we recently described that the visceral fat of cold-exposed humans living in Siberia is mainly formed by a large amount of BAT. We found that 12% of perirenal and 30% of periaortic fat depots are composed by UCP1 immunoreactive BAT. Strikingly in outdoor workers the perirenal BAT almost doubled (22%) and periaortic BAT reached 37% [31].

In conclusion, the demonstration that most fat depots in mice and humans are organized to form a single unitary organ, as also confirmed by the first 3D reconstruction reported in the present paper, provides important insights into the identification of future therapeutic strategies for obesity and related disorders based on the browning of visceral fat [7]. White and brown adipocytes secrete several hormones, many of these influencing eating behavior and energy expenditure [52,53,54]. From this perspective, the adipose organ should also be considered the largest endocrine organ in mammals. Finally, in the mammalian body, anatomical and functional data allow proposing the existence of a new system in which the adipose organ collaborates with the digestive organs to distribute energy between thermogenesis and metabolism: the nutritional system. Considering that the adipocytes of subcutaneous depots significantly contribute to the mammary gland milk production through lipids fueling and reversible adipocyte-epithelial transdifferentiation [27], the nutritional system not only allows short-term homeostasis, but is also arranged to ensure long-term homeostasis satisfying pup nutritional needs through lactation. The increasingly wide adipocytes heterogeneity, documented within each adipose depots thanks to the recent technological advances, probably are more linked to different functional stages than subpopulations, but opens novel questions concerning adipose tissues functional organization of the adipose organ, a topic that deserve future investigation [55,56,57,58].

## Supplementary Materials

The following supporting information can be downloaded at: https://www.mdpi.com/article/10.3390/biomedicines10092275/s1.

## References

[1] Cinti S. (2018). Adipose Organ Development and Remodeling. Compr. Physiol..

[2] Cannon B., Nedergaard J. (2004). Brown adipose tissue: Function and physiological significance. Physiol Rev..

[3] Barbatelli G., Murano I., Madsen L., Hao Q., Jimenez M., Kristiansen K., Giacobino J.P., De Matteis R., Cinti S. (2010). The emergence of cold-induced brown adipocytes in mouse white fat depots is determined predominantly by white to brown adipocyte transdifferentiation. Am. J. Physiol. Endocrinol. Metab..

[4] Altshuler-Keylin S., Shinoda K., Hasegawa Y., Ikeda K., Hong H., Kang Q., Yang Y., Perera R.M., Debnath J., Kajimura S. (2016). Beige Adipocyte Maintenance Is Regulated by Autophagy-Induced Mitochondrial Clearance. Cell Metab..

[5] Kotzbeck P., Giordano A., Mondini E., Murano I., Severi I., Venema W., Cecchini M.P., Kershaw E.E., Barbatelli G., Haemmerle G. (2018). Brown adipose tissue whitening leads to brown adipocyte death and adipose tissue inflammation. J. Lipid Res..

[6] Wells G.S. (1940). Adipose Tissue, A Neglected Subject. JAMA.

[7] Giordano A., Frontini A., Cinti S. (2016). Convertible visceral fat as a therapeutic target to curb obesity. Nat. Rev. Drug Discov..

[8] Himms-Hagen J., Cui J., Danforth E., Taatjes D.J., Lang S.S., Waters B.L., Claus T.H. (1994). Effect of CL-316,243, a thermogenic beta 3-agonist, on energy balance and brown and white adipose tissues in rats. Am. J. Physiol..

[9] Collins S., Daniel K.W., Petro A.E., Surwit R.S. (1997). Strain-specific response to beta 3-adrenergic receptor agonist treatment of diet-induced obesity in mice. Endocrinology.

[10] Ghorbani M., Himms-Hagen J. (1997). Appearance of brown adipocytes in white adipose tissue during CL 316,243-induced reversal of obesity and diabetes in Zucker fa/fa rats. Int. J. Obes. Relat. Metab. Disord..

[11] Fisher M.H., Amend A.M., Bach T.J., Barker J.M., Brady E.J., Candelore M.R., Carroll D., Cascieri M.A., Chiu S.H., Deng L. (1998). A selective human beta3 adrenergic receptor agonist increases metabolic rate in rhesus monkeys. J. Clin. Investig..

[12] Sasaki N., Uchida E., Niiyama M., Yoshida T., Saito M. (1998). Anti-obesity effects of selective agonists to the beta 3-adrenergic receptor in dogs. I. The presence of canine beta 3-adrenergic receptor and in vivo lipomobilization by its agonists. J. Vet. Med. Sci..

[13] O'Mara A.E., Johnson J.W., Linderman J.D., Brychta R.J., McGehee S., Fletcher L.A., Fink Y.A., Kapuria D., Cassimatis T.M., Kelsey N. (2020). Chronic mirabegron treatment increases human brown fat, HDL cholesterol, and insulin sensitivity. J. Clin. Investig..

[14] Bowman K., Atkins J.L., Delgado J., Kos K., Kuchel G.A., Ble A., Ferrucci L., Melzer D. (2017). Central adiposity and the overweight risk paradox in aging: Follow-up of 130,473 UK Biobank participants. Am. J. Clin. Nutr..

[15] Cypess A.M., Weiner L.S., Roberts-Toler C., Franquet Elia E., Kessler S.H., Kahn P.A., English J., Chatman K., Trauger S.A., Doria A. (2015). Activation of human brown adipose tissue by a beta3-adrenergic receptor agonist. Cell Metab..

[16] Kuji I., Imabayashi E., Minagawa A., Matsuda H., Miyauchi T. (2008). Brown adipose tissue demonstrating intense FDG uptake in a patient with mediastinal pheochromocytoma. Ann. Nucl. Med..

[17] Saito M., Okamatsu-Ogura Y., Matsushita M., Watanabe K., Yoneshiro T., Nio-Kobayashi J., Iwanaga T., Miyagawa M., Kameya T., Nakada K. (2009). High incidence of metabolically active brown adipose tissue in healthy adult humans: Effects of cold exposure and adiposity. Diabetes.

[18] Colleluori G., Perugini J., Di Vincenzo A., Senzacqua M., Giordano A., Cinti S. (2022). Brown Fat Anatomy in Humans and Rodents. Methods Mol. Biol..

[19] Spitzer V., Ackerman M.J., Scherzinger A.L., Whitlock D. (1996). The visible human male: A technical report. J. Am. Med. Inform. Assoc..

[20] Ackerman M.J. (2017). The Visible Human Project: From Body to Bits. IEEE Pulse.

[21] Park J.S., Chung M.S., Hwang S.B., Lee Y.S., Har D.H., Park H.S. (2005). Visible Korean human: Improved serially sectioned images of the entire body. IEEE Trans. Med. Imaging.

[22] Chung B.S., Park H.S., Park J.S., Hwang S.B., Chung M.S. (2021). Sectioned and segmented images of the male whole body, female whole body, male head, and female pelvis from the Visible Korean. Anat. Sci. Int..

[23] Paech D., Giesel F.L., Unterhinninghofen R., Schlemmer H.P., Kuner T., Doll S. (2017). Cadaver-specific CT scans visualized at the dissection table combined with virtual dissection tables improve learning performance in general gross anatomy. Eur. Radiol..

[24] Ward T.M., Wertz C.I., Mickelsen W. (2018). Anatomage Table Enhances Radiologic Technology Education. Radiol. Technol..

[25] Delfaut E.M., Beltran J., Johnson G., Rousseau J., Marchandise X., Cotten A. (1999). Fat suppression in MR imaging: Techniques and pitfalls. Radiographics.

[26] Neville M.C., Medina D., Monks J., Hovey R.C. (1998). The mammary fat pad. J. Mammary Gland Biol. Neoplasia.

[27] Colleluori G., Perugini J., Barbatelli G., Cinti S. (2021). Mammary gland adipocytes in lactation cycle, obesity and breast cancer. Rev. Endocr. Metab. Disord..

[28] Thomas E.L., Saeed N., Hajnal J.V., Brynes A., Goldstone A.P., Frost G., Bell J.D. (1998). Magnetic resonance imaging of total body fat. J. Appl. Physiol. (1985).

[29] Kim Y.J., Lee S.H., Kim T.Y., Park J.Y., Choi S.H., Kim K.G. (2013). Body fat assessment method using CT images with separation mask algorithm. J. Digit. Imaging.

[30] Hu H.H., Kim H.W., Nayak K.S., Goran M.I. (2010). Comparison of fat-water MRI and single-voxel MRS in the assessment of hepatic and pancreatic fat fractions in humans. Obesity.

[31] Efremova A., Senzacqua M., Venema W., Isakov E., Di Vincenzo A., Zingaretti M.C., Protasoni M., Thomski M., Giordano A., Cinti S. (2019). A large proportion of mediastinal and perirenal visceral fat of Siberian adult people is formed by UCP1 immunoreactive multilocular and paucilocular adipocytes. J. Physiol. Biochem..

[32] Bluher M. (2019). Obesity: Global epidemiology and pathogenesis. Nat. Rev. Endocrinol.

[33] Frank J. (2016). Origins of the obesity pandemic can be analysed. Nature.

[34] EASO (2020). Obesity Statistics. https://easo.org/media-portal/statistics/;.

[35] Muller T.D., Bluher M., Tschop M.H., DiMarchi R.D. (2022). Anti-obesity drug discovery: Advances and challenges. Nat. Rev. Drug Discov..

[36] Wang Q.A., Tao C., Gupta R.K., Scherer P.E. (2013). Tracking adipogenesis during white adipose tissue development, expansion and regeneration. Nat. Med..

[37] Jeffery E., Church C.D., Holtrup B., Colman L., Rodeheffer M.S. (2015). Rapid depot-specific activation of adipocyte precursor cells at the onset of obesity. Nat. Cell Biol..

[38] Cinti S., Mitchell G., Barbatelli G., Murano I., Ceresi E., Faloia E., Wang S., Fortier M., Greenberg A.S., Obin M.S. (2005). Adipocyte death defines macrophage localization and function in adipose tissue of obese mice and humans. J. Lipid Res..

[39] Giordano A., Murano I., Mondini E., Perugini J., Smorlesi A., Severi I., Barazzoni R., Scherer P.E., Cinti S. (2013). Obese adipocytes show ultrastructural features of stressed cells and die of pyroptosis. J. Lipid Res..

[40] Weisberg S.P., McCann D., Desai M., Rosenbaum M., Leibel R.L., Ferrante A.W. (2003). Obesity is associated with macrophage accumulation in adipose tissue. J. Clin. Investig..

[41] Xu H., Barnes G.T., Yang Q., Tan G., Yang D., Chou C.J., Sole J., Nichols A., Ross J.S., Tartaglia L.A. (2003). Chronic inflammation in fat plays a crucial role in the development of obesity-related insulin resistance. J. Clin. Investig..

[42] Belligoli A., Compagnin C., Sanna M., Favaretto F., Fabris R., Busetto L., Foletto M., Dal Pra C., Serra R., Prevedello L. (2019). Characterization of subcutaneous and omental adipose tissue in patients with obesity and with different degrees of glucose impairment. Sci. Rep..

[43] American Diabetes A. (2021). Obesity Management for the Treatment of Type 2 Diabetes: Standards of Medical Care in Diabetes-2021. Diabetes Care.

[44] Vijgen G.H., Bouvy N.D., Teule G.J., Brans B., Schrauwen P., van Marken Lichtenbelt W.D. (2011). Brown adipose tissue in morbidly obese subjects. PLoS ONE.

[45] Cypess A.M., Lehman S., Williams G., Tal I., Rodman D., Goldfine A.B., Kuo F.C., Palmer E.L., Tseng Y.H., Doria A. (2009). Identification and importance of brown adipose tissue in adult humans. N. Engl. J. Med..

[46] van Marken Lichtenbelt W.D., Vanhommerig J.W., Smulders N.M., Drossaerts J.M., Kemerink G.J., Bouvy N.D., Schrauwen P., Teule G.J. (2009). Cold-activated brown adipose tissue in healthy men. N. Engl. J. Med..

[47] Virtanen K.A., Lidell M.E., Orava J., Heglind M., Westergren R., Niemi T., Taittonen M., Laine J., Savisto N.J., Enerback S. (2009). Functional brown adipose tissue in healthy adults. N. Engl J. Med..

[48] Rosenwald M., Perdikari A., Rulicke T., Wolfrum C. (2013). Bi-directional interconversion of brite and white adipocytes. Nat. Cell Biol..

[49] Chondronikola M., Volpi E., Borsheim E., Porter C., Annamalai P., Enerback S., Lidell M.E., Saraf M.K., Labbe S.M., Hurren N.M. (2014). Brown adipose tissue improves whole-body glucose homeostasis and insulin sensitivity in humans. Diabetes.

[50] Berbee J.F., Boon M.R., Khedoe P.P., Bartelt A., Schlein C., Worthmann A., Kooijman S., Hoeke G., Mol I.M., John C. (2015). Brown fat activation reduces hypercholesterolaemia and protects from atherosclerosis development. Nat. Commun..

[51] Seale P., Conroe H.M., Estall J., Kajimura S., Frontini A., Ishibashi J., Cohen P., Cinti S., Spiegelman B.M. (2011). Prdm16 determines the thermogenic program of subcutaneous white adipose tissue in mice. J. Clin. Investig..

[52] Villarroya F., Cereijo R., Villarroya J., Giralt M. (2017). Brown adipose tissue as a secretory organ. Nat. Rev. Endocrinol..

[53] Zhang Y., Proenca R., Maffei M., Barone M., Leopold L., Friedman J.M. (1994). Positional cloning of the mouse obese gene and its human homologue. Nature.

[54] Romere C., Duerrschmid C., Bournat J., Constable P., Jain M., Xia F., Saha P.K., Del Solar M., Zhu B., York B. (2016). Asprosin, a Fasting-Induced Glucogenic Protein Hormone. Cell.

[55] Sun W.D.H., Balaz M., Slyper M., Drokhlyansky E., Colleluori G., Giordano A., Kovanicova Z., Stefanicka P., Ding L., Rudofsky G. (2020). Single-nucleus RNA-Seq Reveals a new type of brown adipocyte regulating thermogenesis. Nature.

[56] Emont M.P., Jacobs C., Essene A.L., Pant D., Tenen D., Colleluori G., Di Vincenzo A., Jorgensen A.M., Dashti H., Stefek A. (2022). A single-cell atlas of human and mouse white adipose tissue. Nature.

[57] Chen Y., Ikeda K., Yoneshiro T., Scaramozza A., Tajima K., Wang Q., Kim K., Shinoda K., Sponton C.H., Brown Z. (2019). Thermal stress induces glycolytic beige fat formation via a myogenic state. Nature.

[58] Cohen P., Kajimura S. (2021). The cellular and functional complexity of thermogenic fat. Nat. Reviews. Mol. Cell Biol..

