# Supplementary figures and images for: The Adipose Organ Is a Unitary Structure in Mice and Humans
Source: Biomedicines. 2022 Sep 14;10(9):2275. doi: 10.3390/biomedicines10092275 (PMC9496043; doi:10.3390/biomedicines10092275)

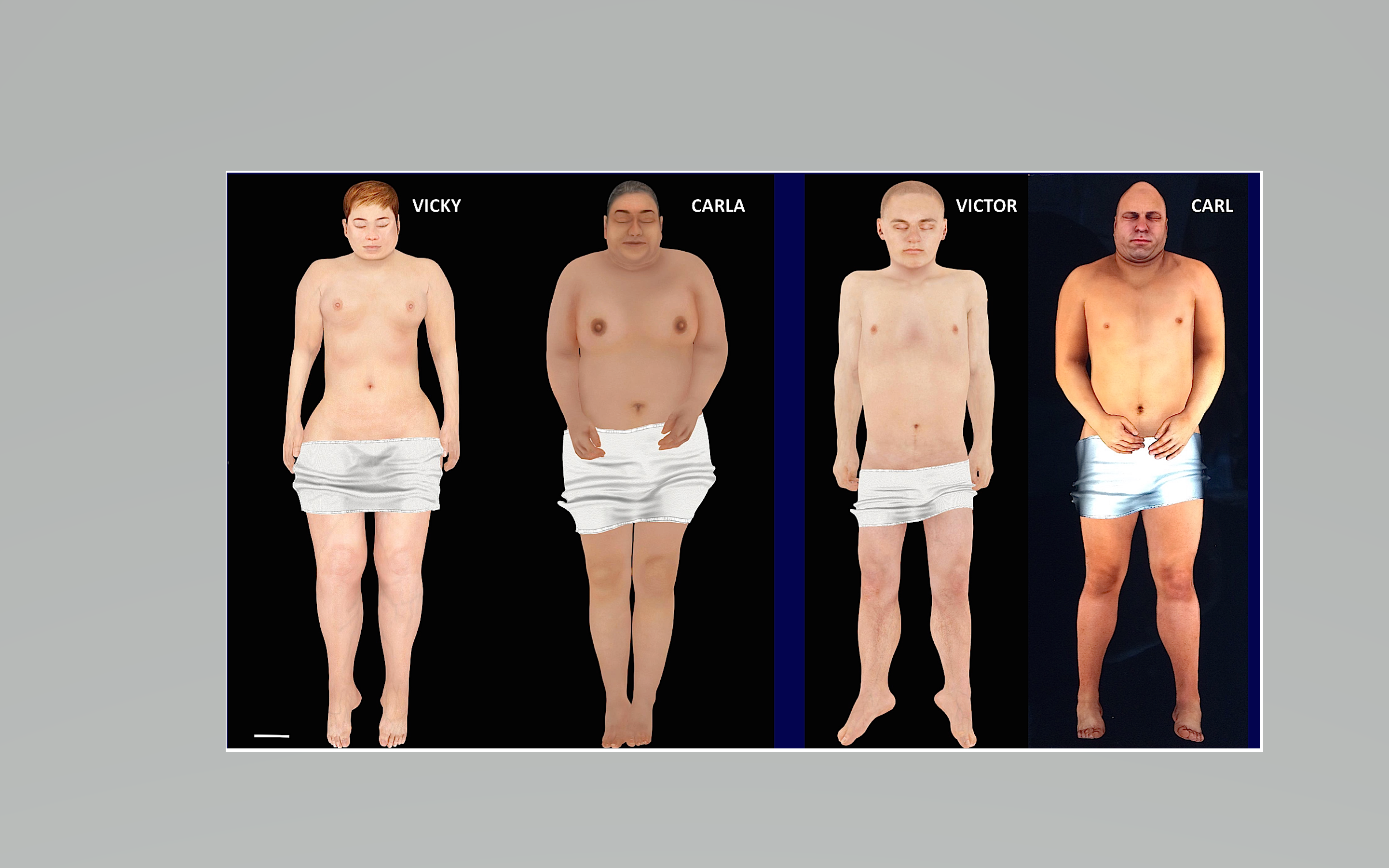

Supplement: Supplementary file 1 [file biomedicines-10-02275-s001.zip › Figure S1.tif]

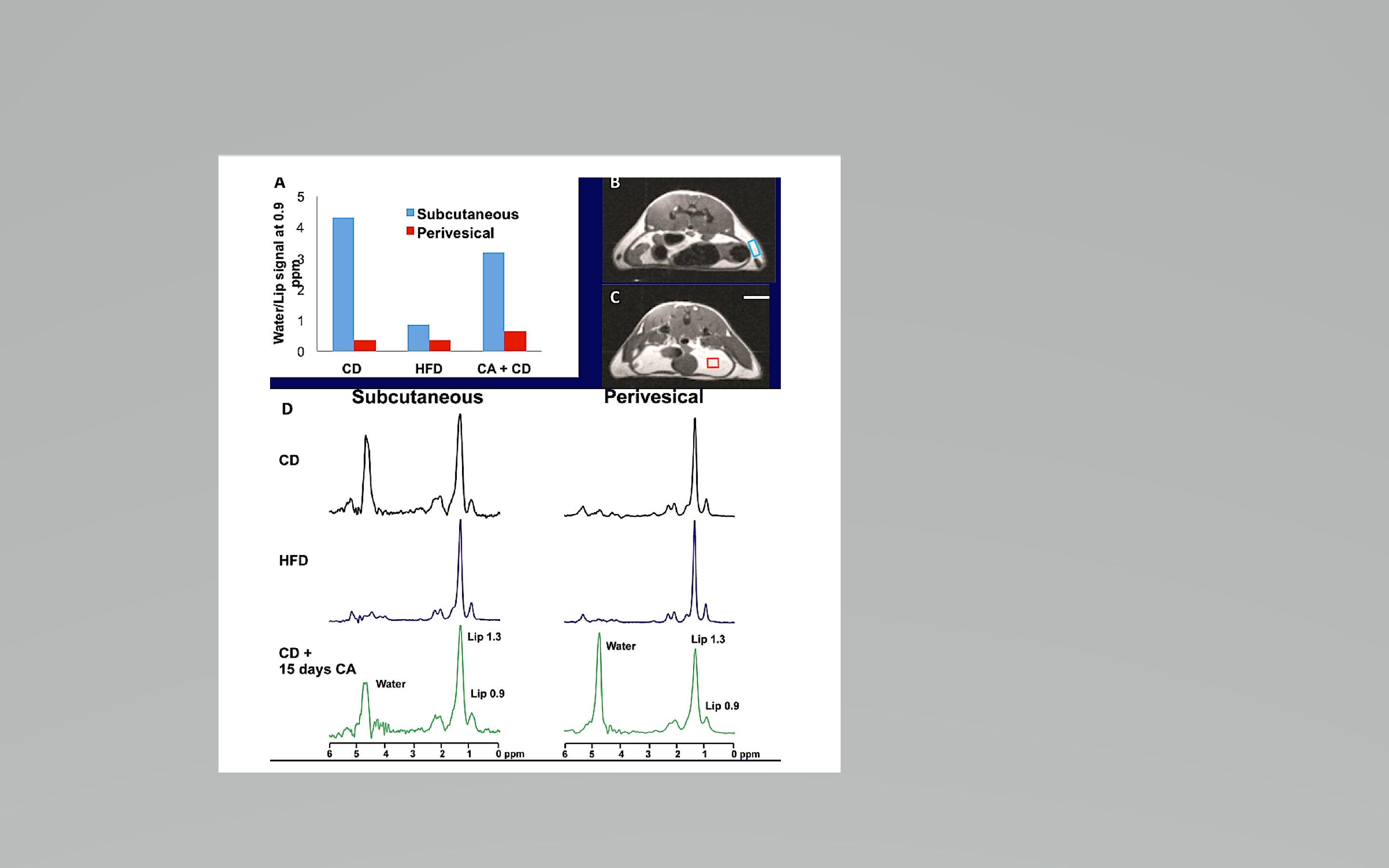

Supplement: Supplementary file 1 [file biomedicines-10-02275-s001.zip › Figure S10.tif]

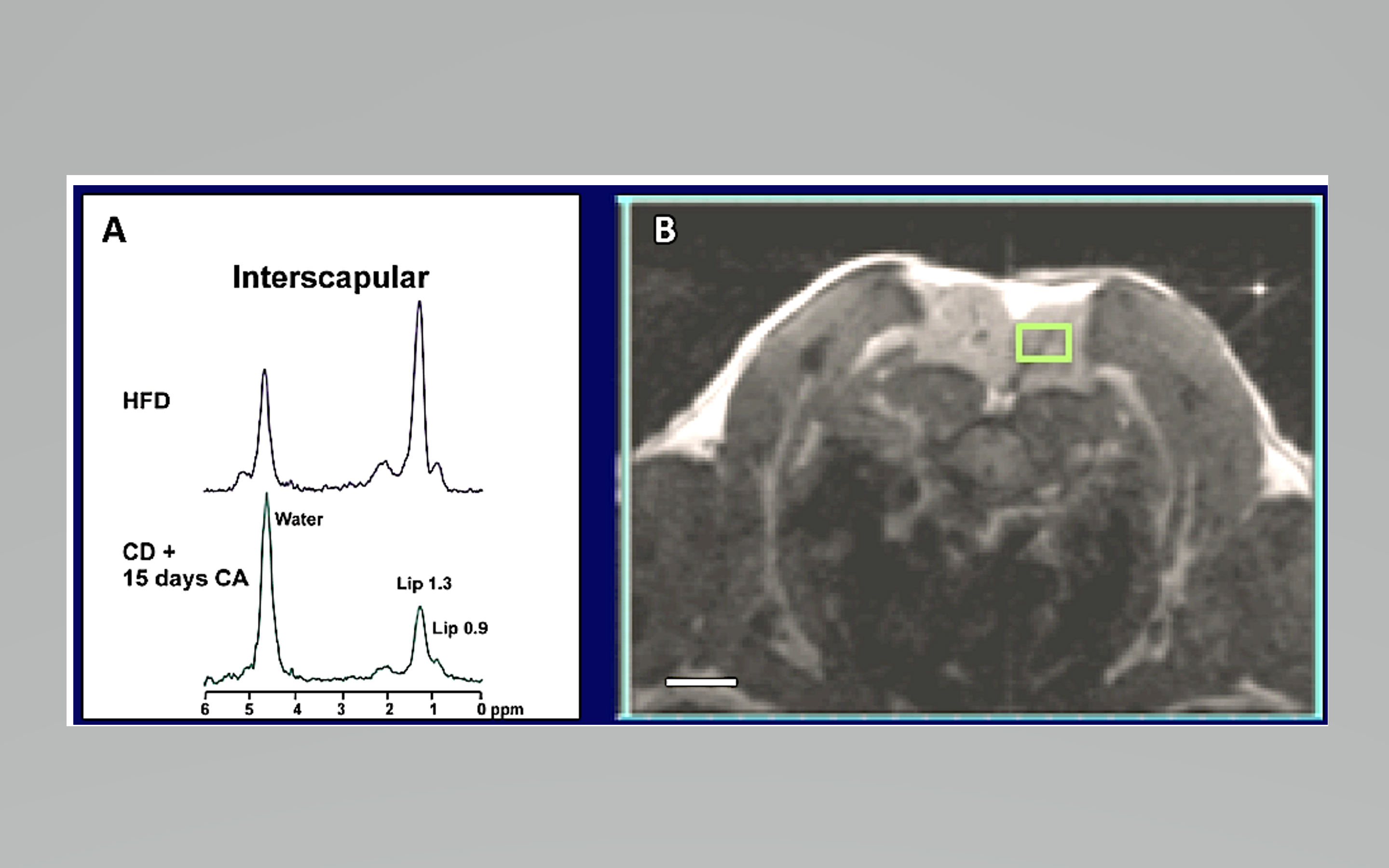

Supplement: Supplementary file 1 [file biomedicines-10-02275-s001.zip › Figure S11.tif]

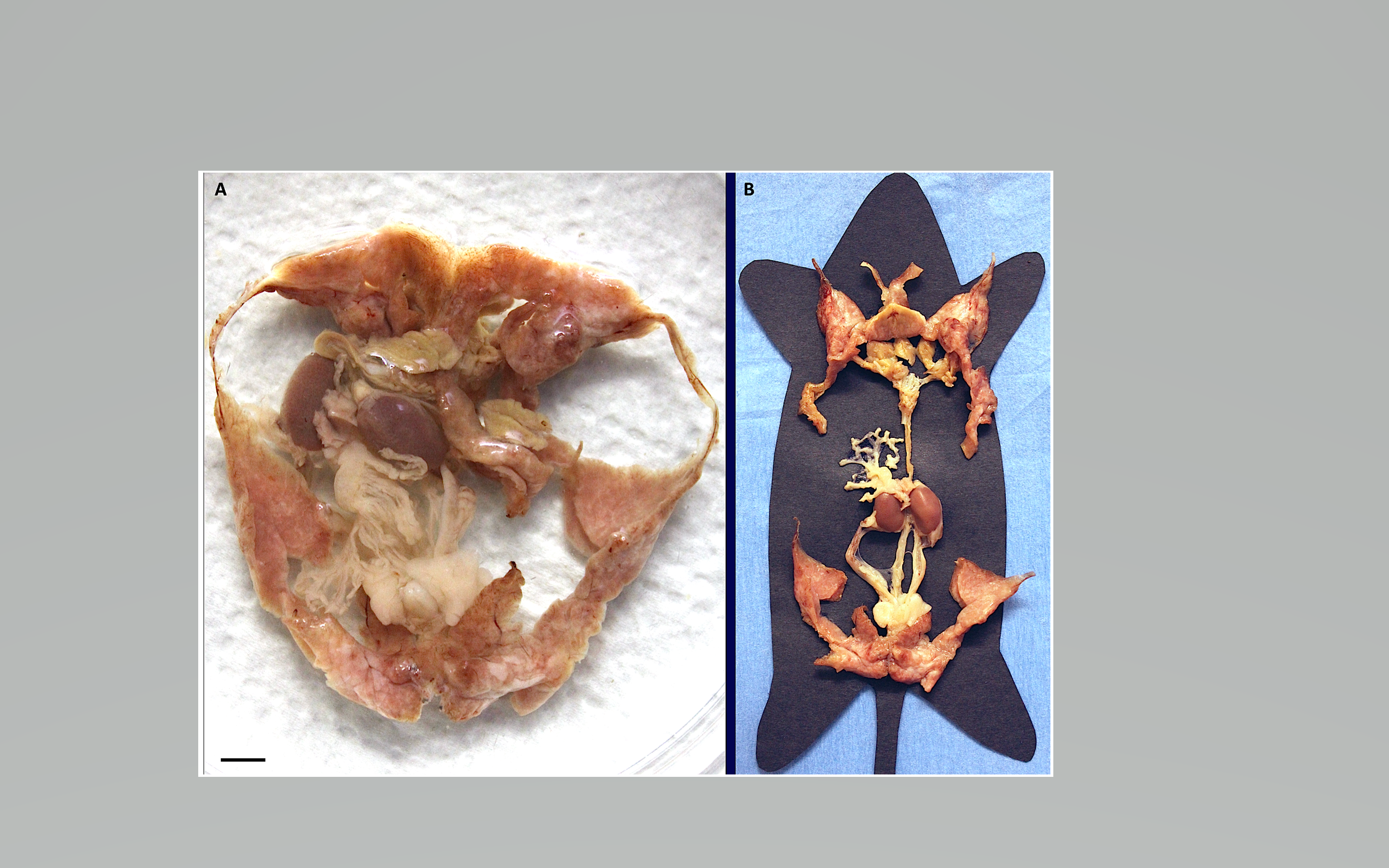

Supplement: Supplementary file 1 [file biomedicines-10-02275-s001.zip › Figure S12.tif]

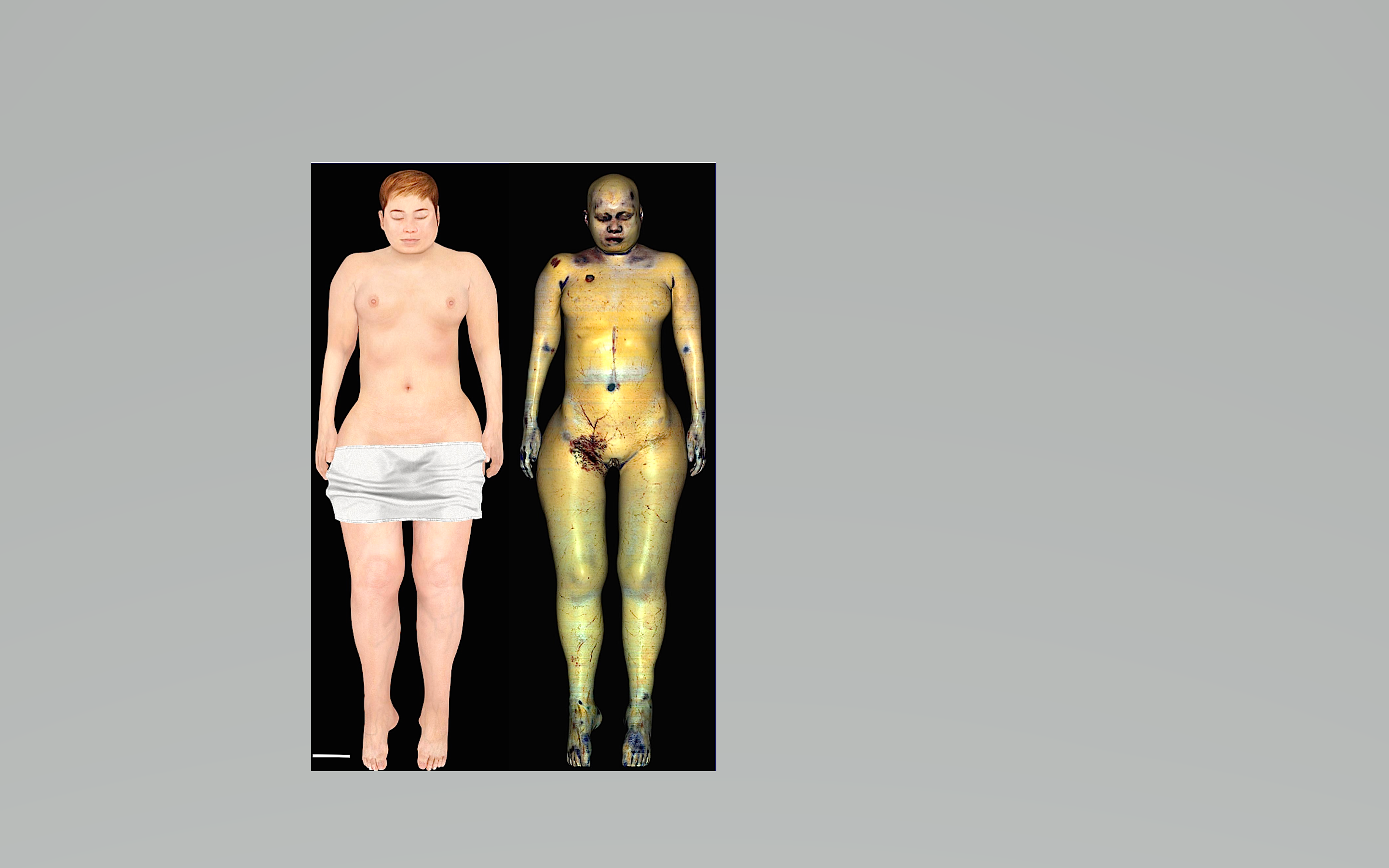

Supplement: Supplementary file 1 [file biomedicines-10-02275-s001.zip › Figure S13.tif]

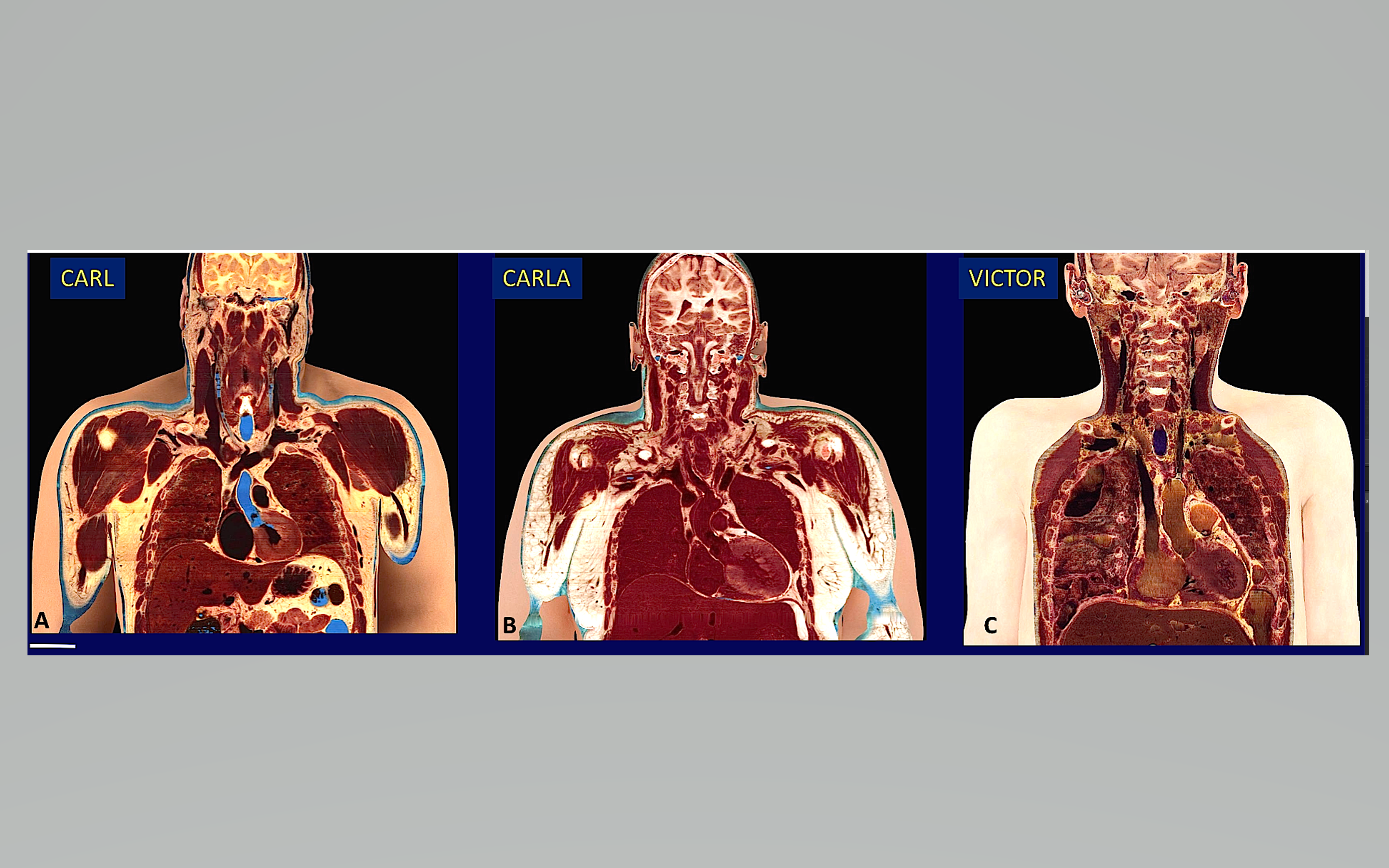

Supplement: Supplementary file 1 [file biomedicines-10-02275-s001.zip › Figure S14.tif]

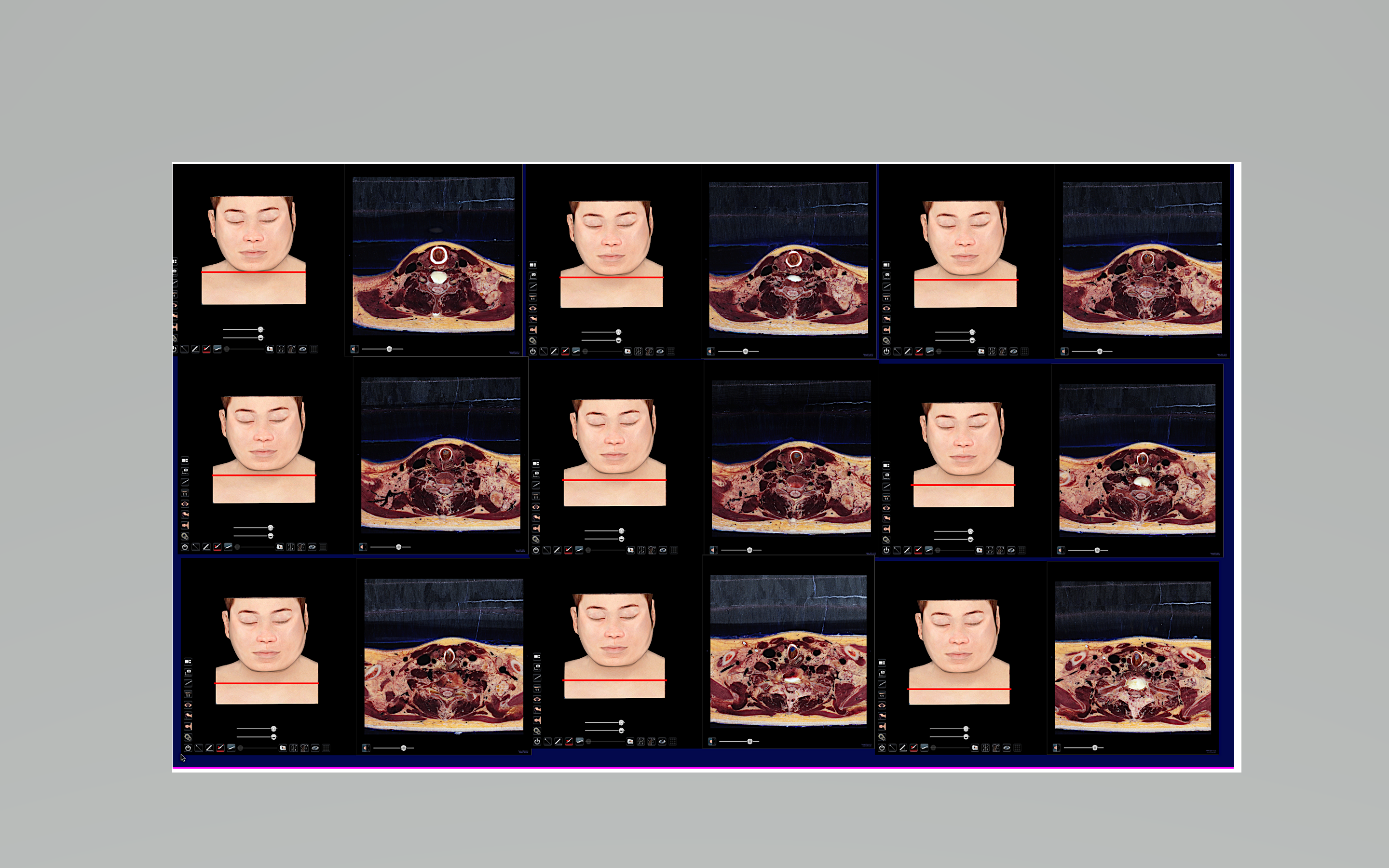

Supplement: Supplementary file 1 [file biomedicines-10-02275-s001.zip › Figure S15.tif]

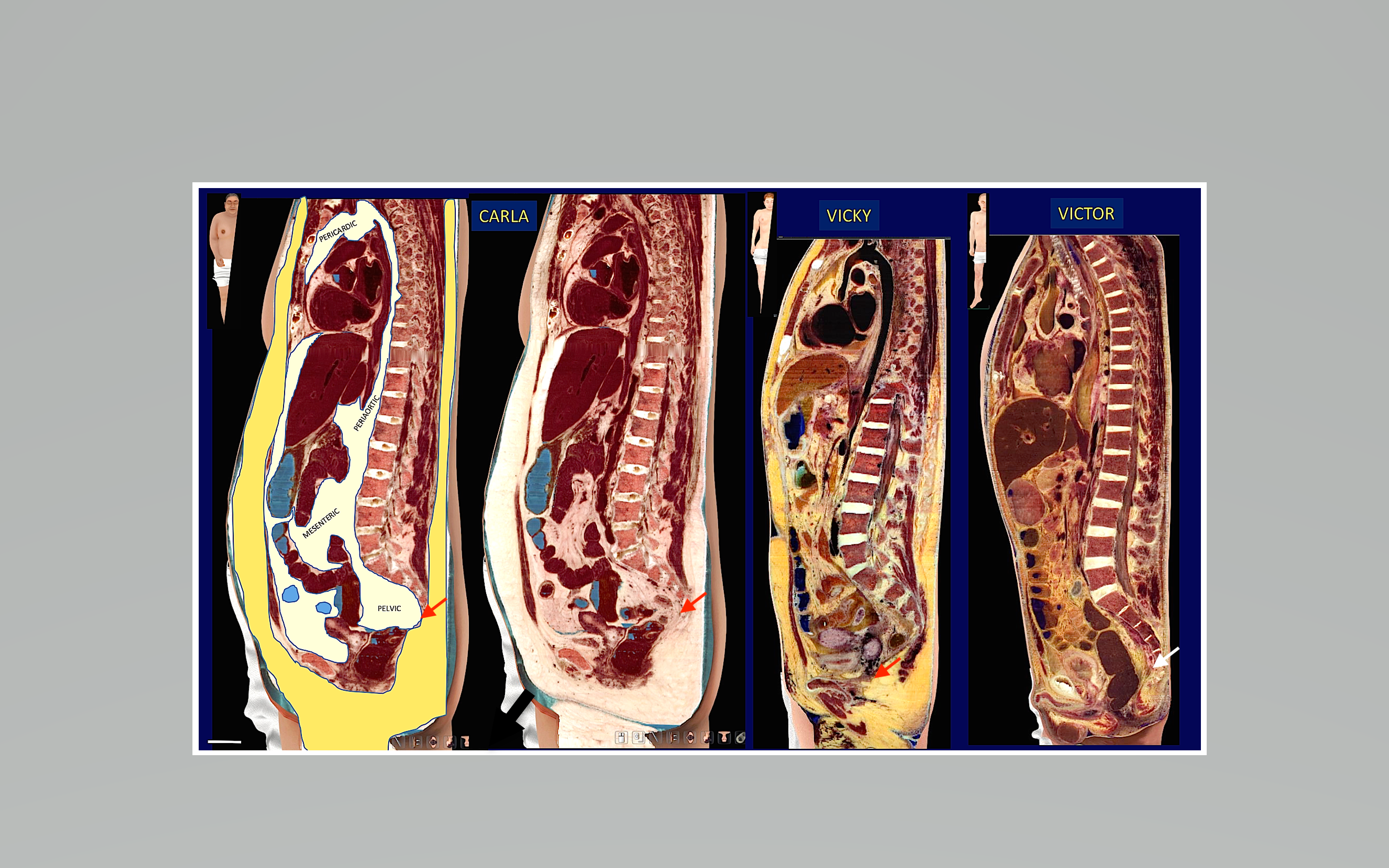

Supplement: Supplementary file 1 [file biomedicines-10-02275-s001.zip › Figure S16.tif]

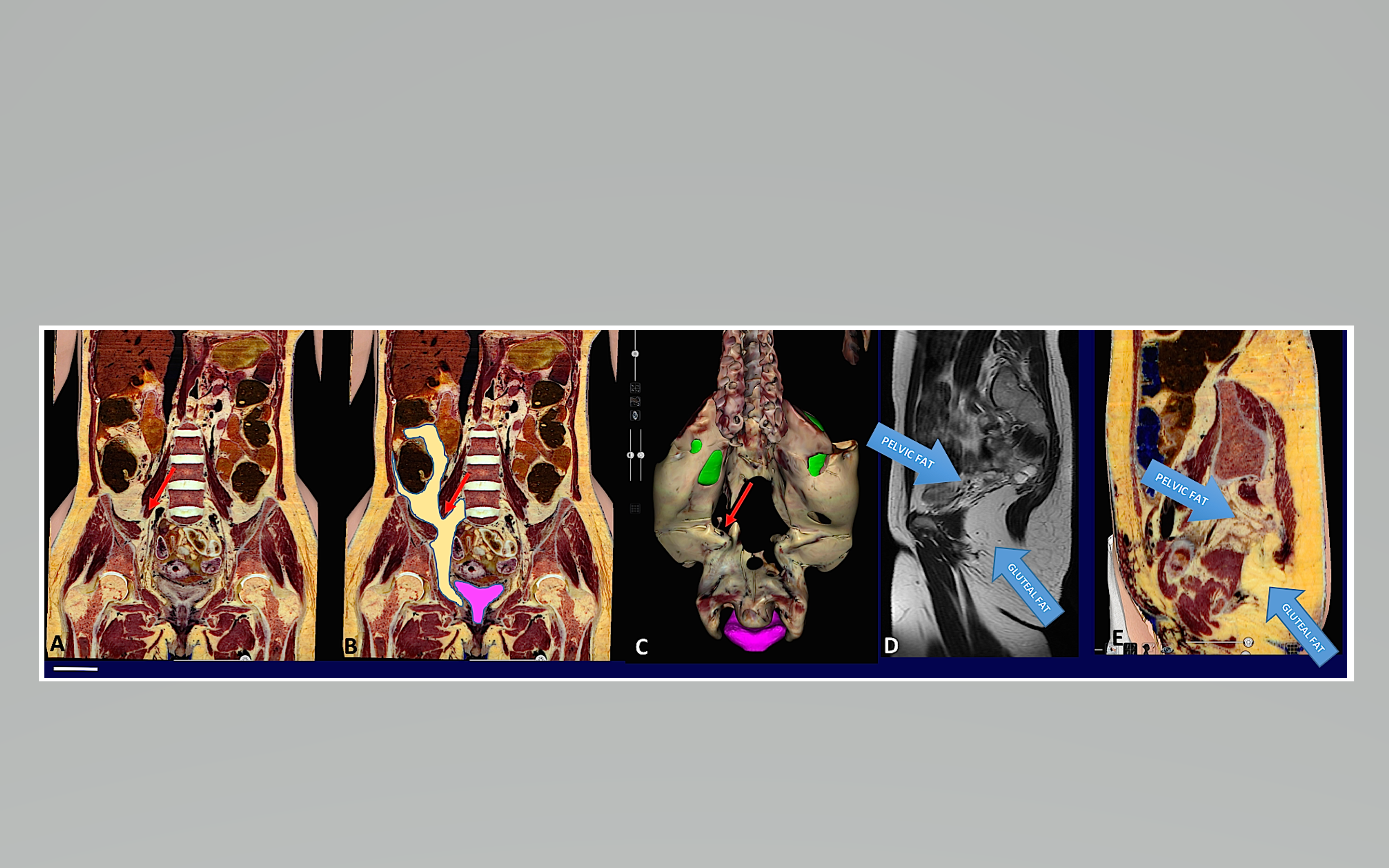

Supplement: Supplementary file 1 [file biomedicines-10-02275-s001.zip › Figure S17.tif]

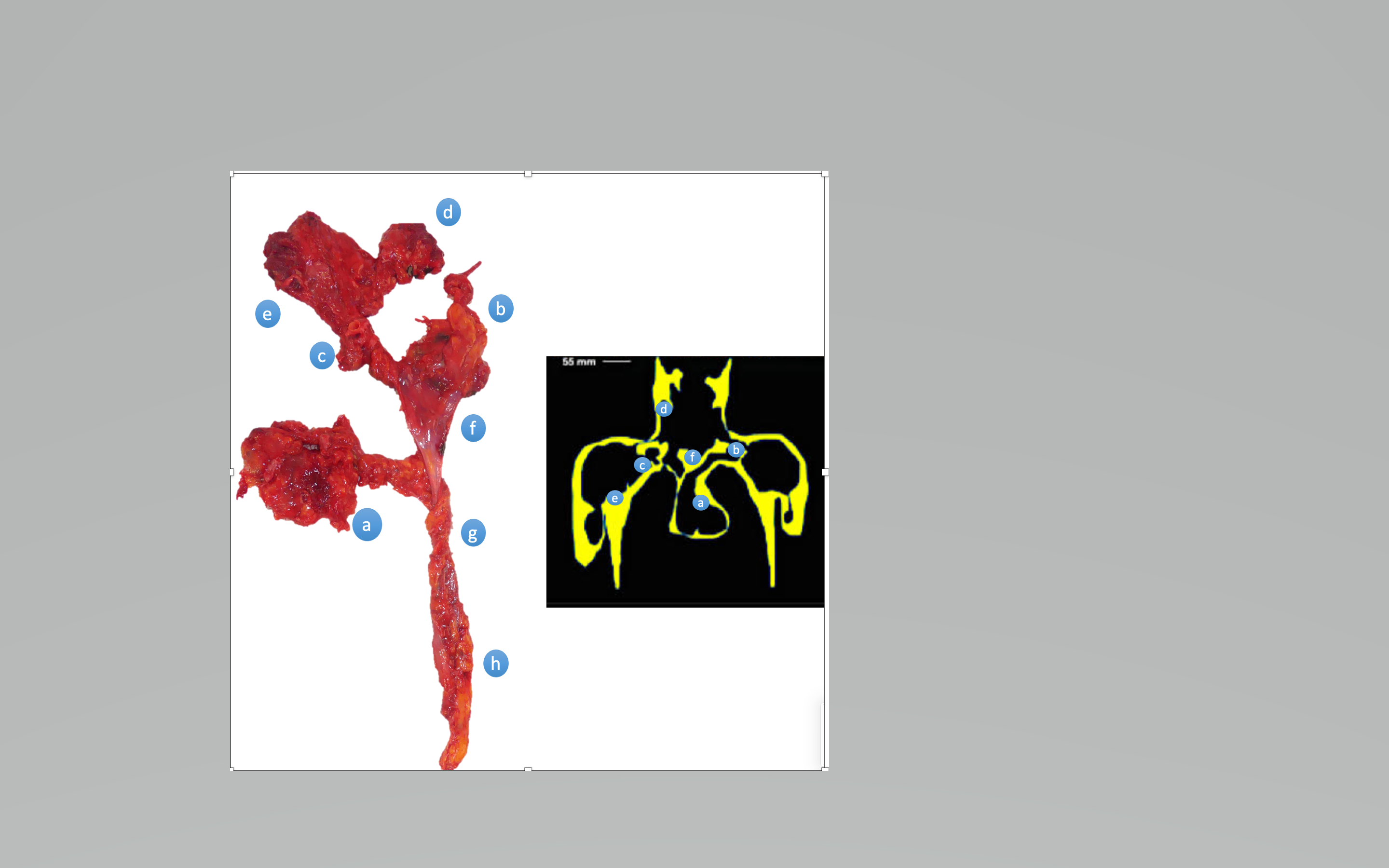

Supplement: Supplementary file 1 [file biomedicines-10-02275-s001.zip › Figure S18.tif]

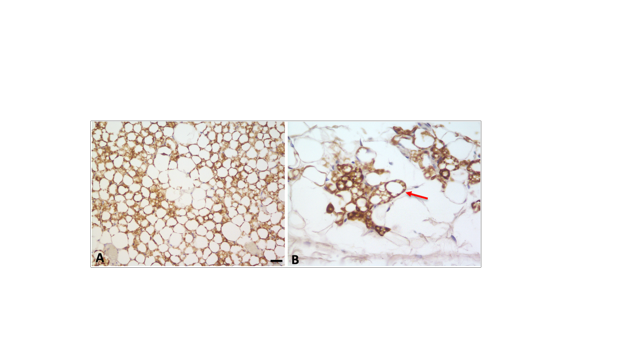

Supplement: Supplementary file 1 [file biomedicines-10-02275-s001.zip › Figure S19.tiff]

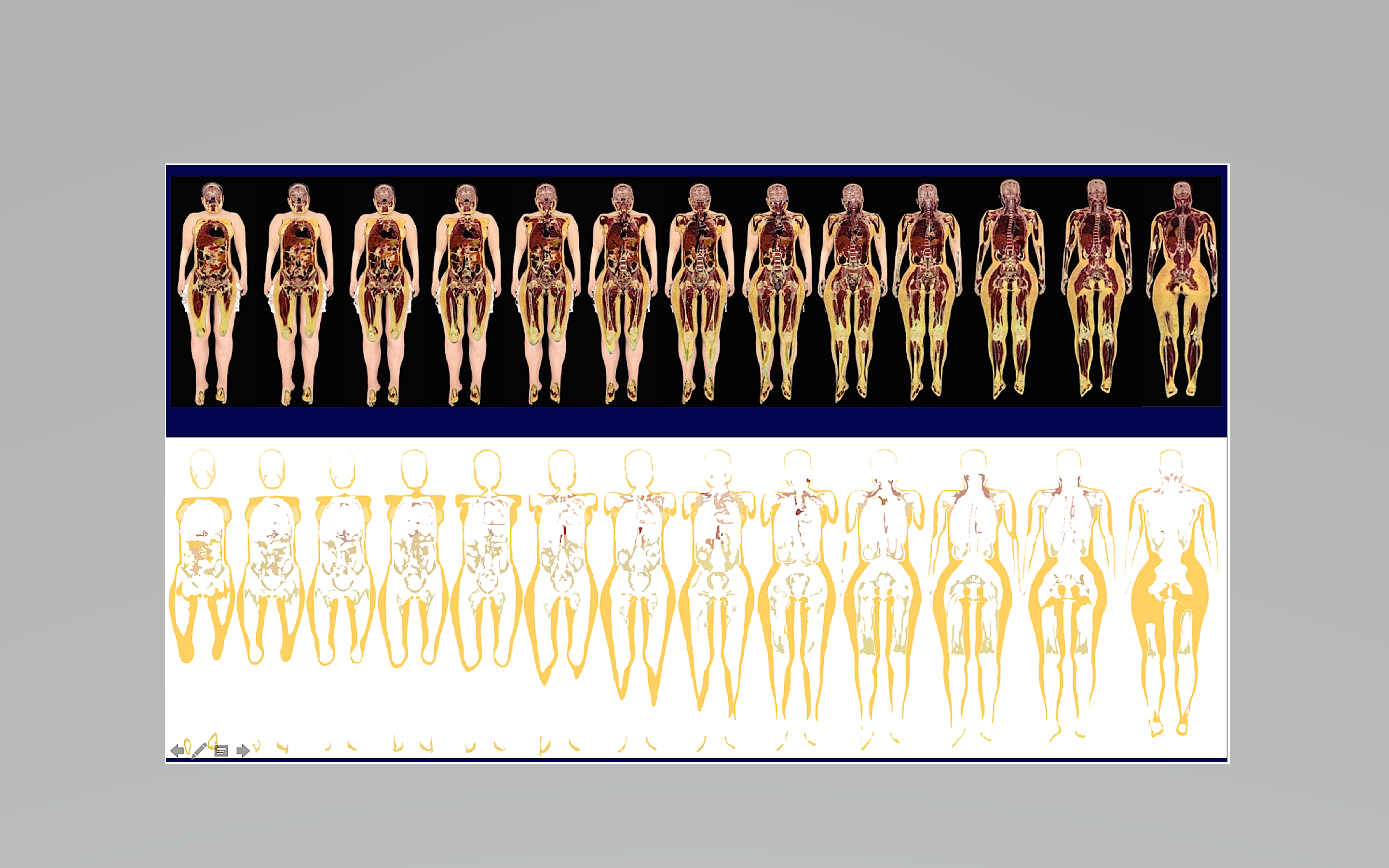

Supplement: Supplementary file 1 [file biomedicines-10-02275-s001.zip › Figure S2.tif]

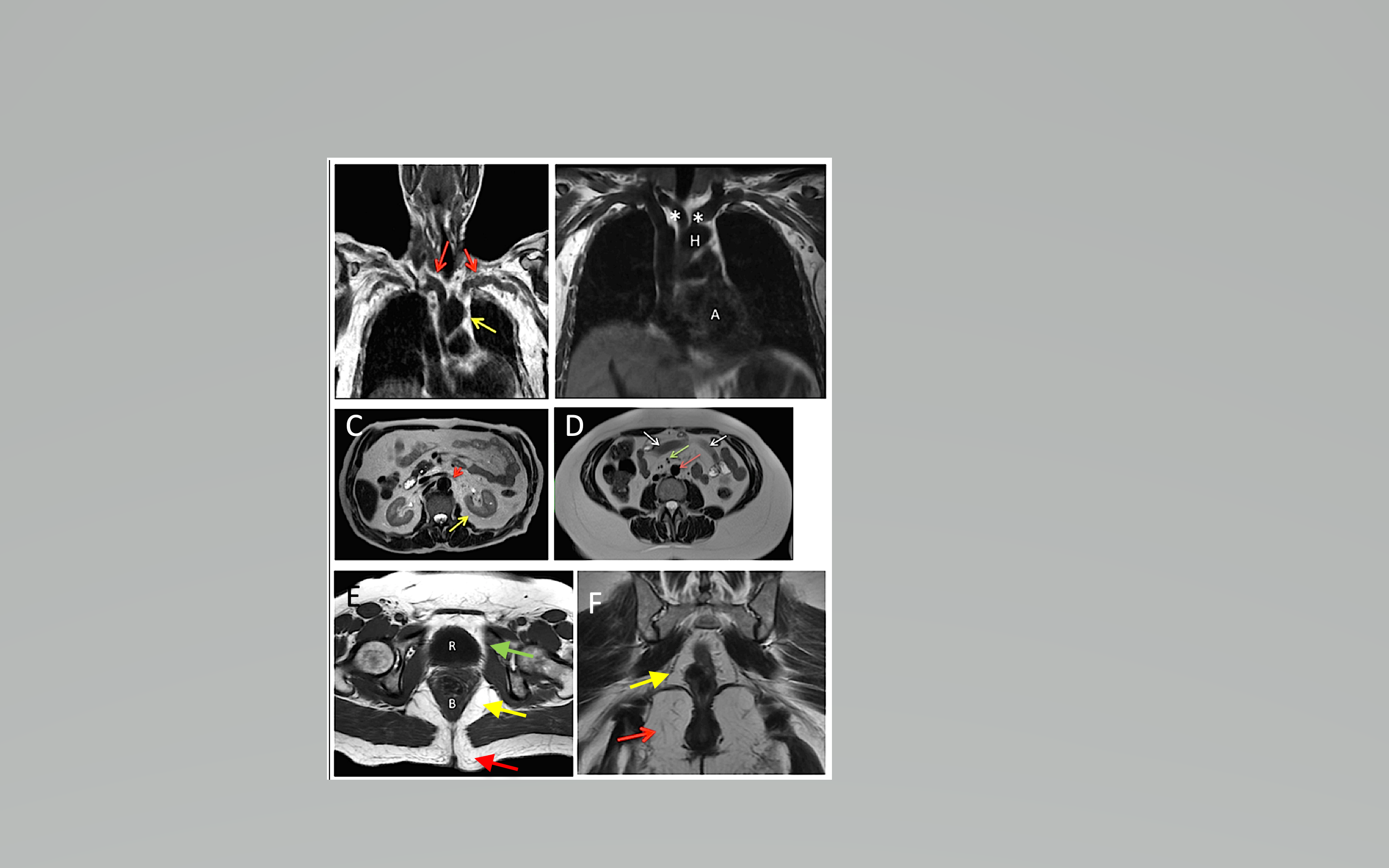

Supplement: Supplementary file 1 [file biomedicines-10-02275-s001.zip › Figure S20.tif]

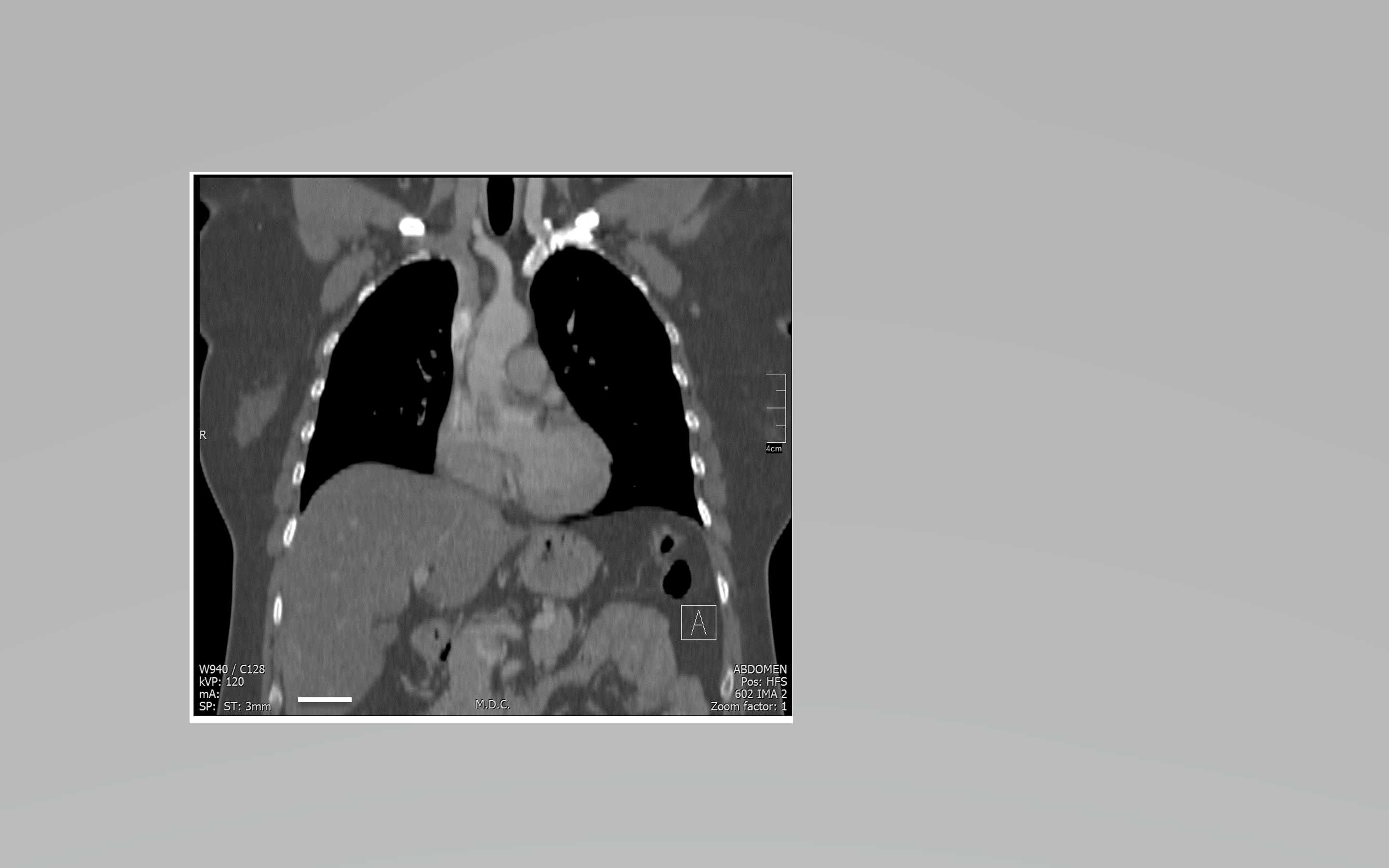

Supplement: Supplementary file 1 [file biomedicines-10-02275-s001.zip › Figure S21.tif]

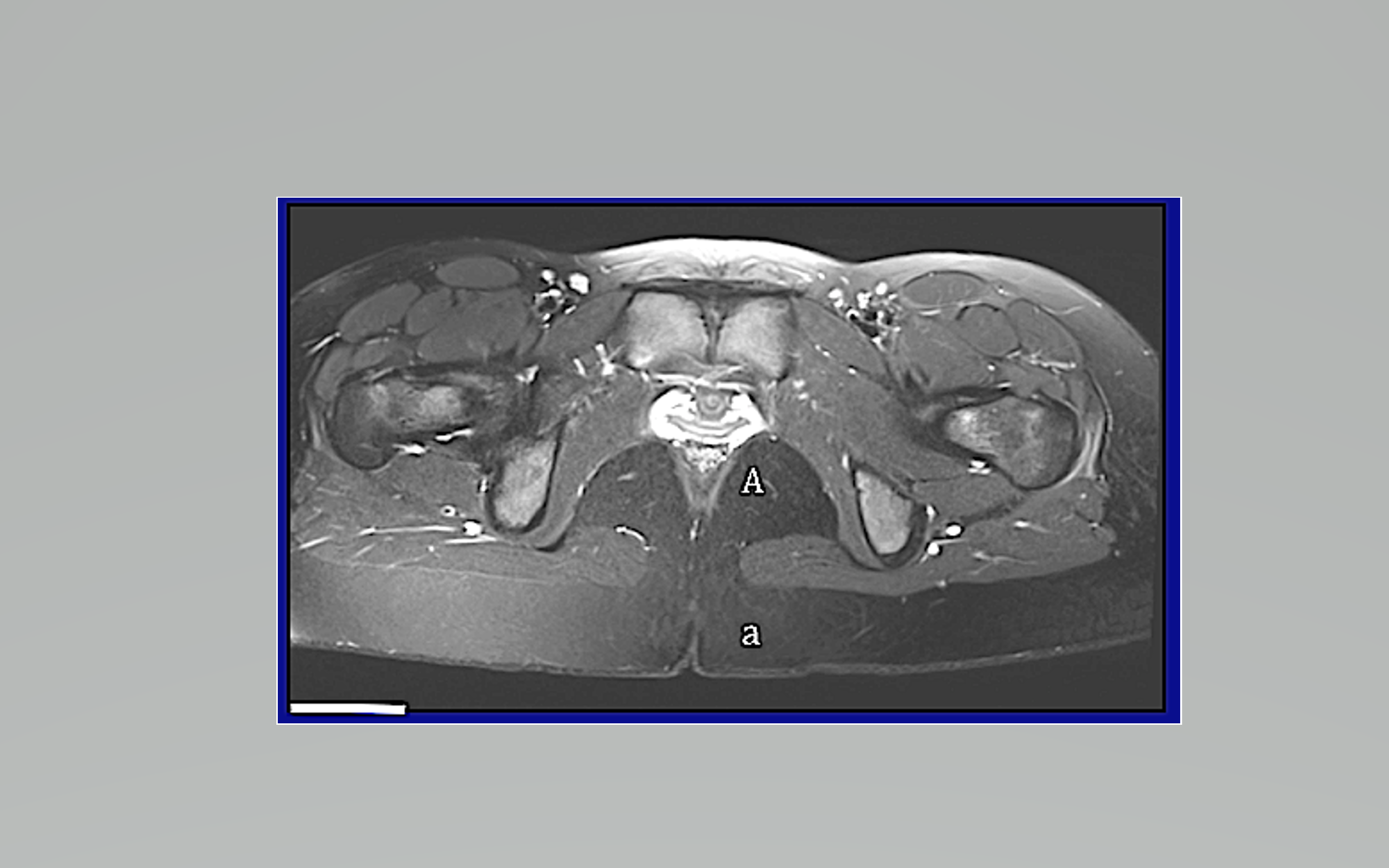

Supplement: Supplementary file 1 [file biomedicines-10-02275-s001.zip › Figure S3.tif]

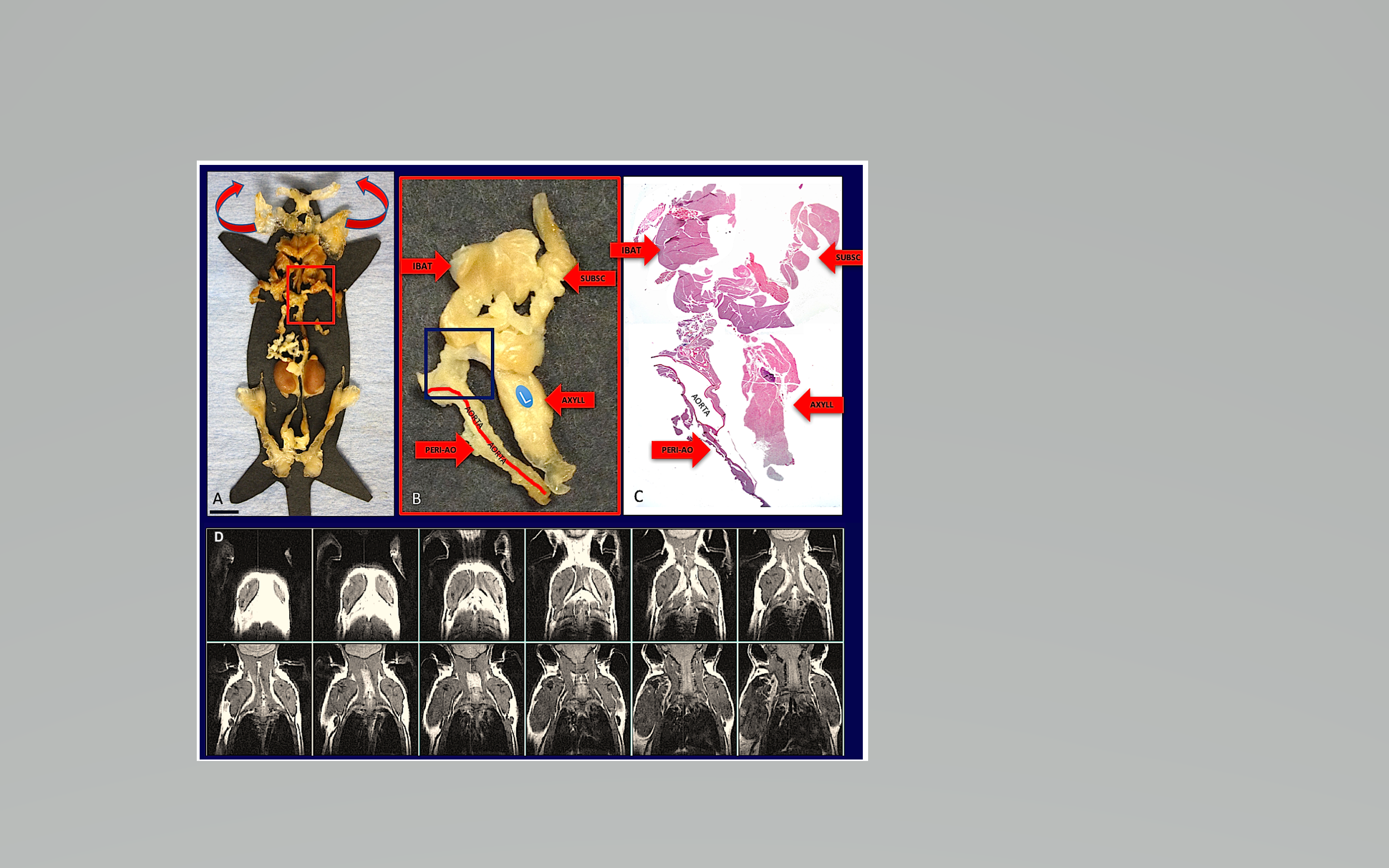

Supplement: Supplementary file 1 [file biomedicines-10-02275-s001.zip › Figure S4.tif]

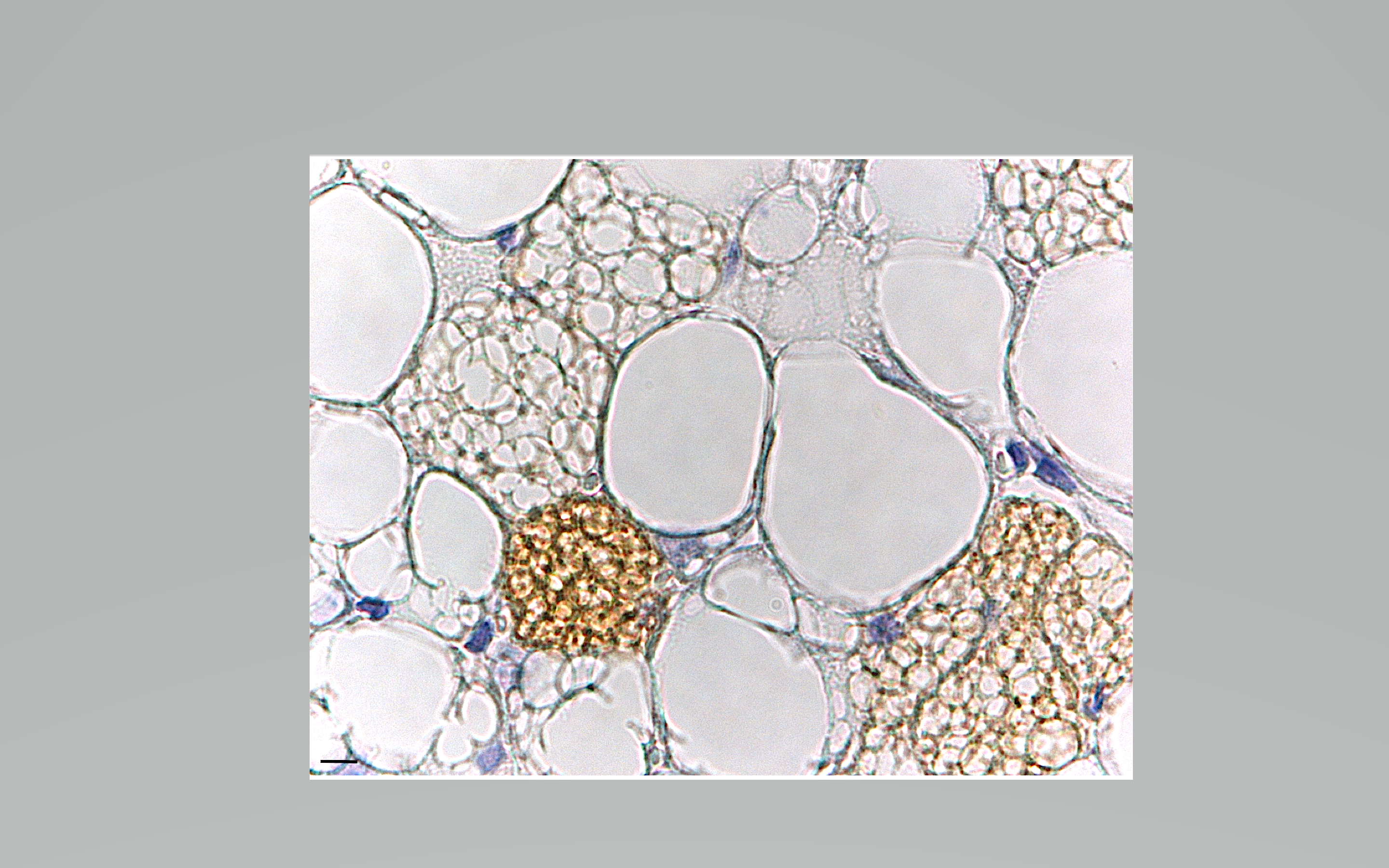

Supplement: Supplementary file 1 [file biomedicines-10-02275-s001.zip › Figure S5.tif]

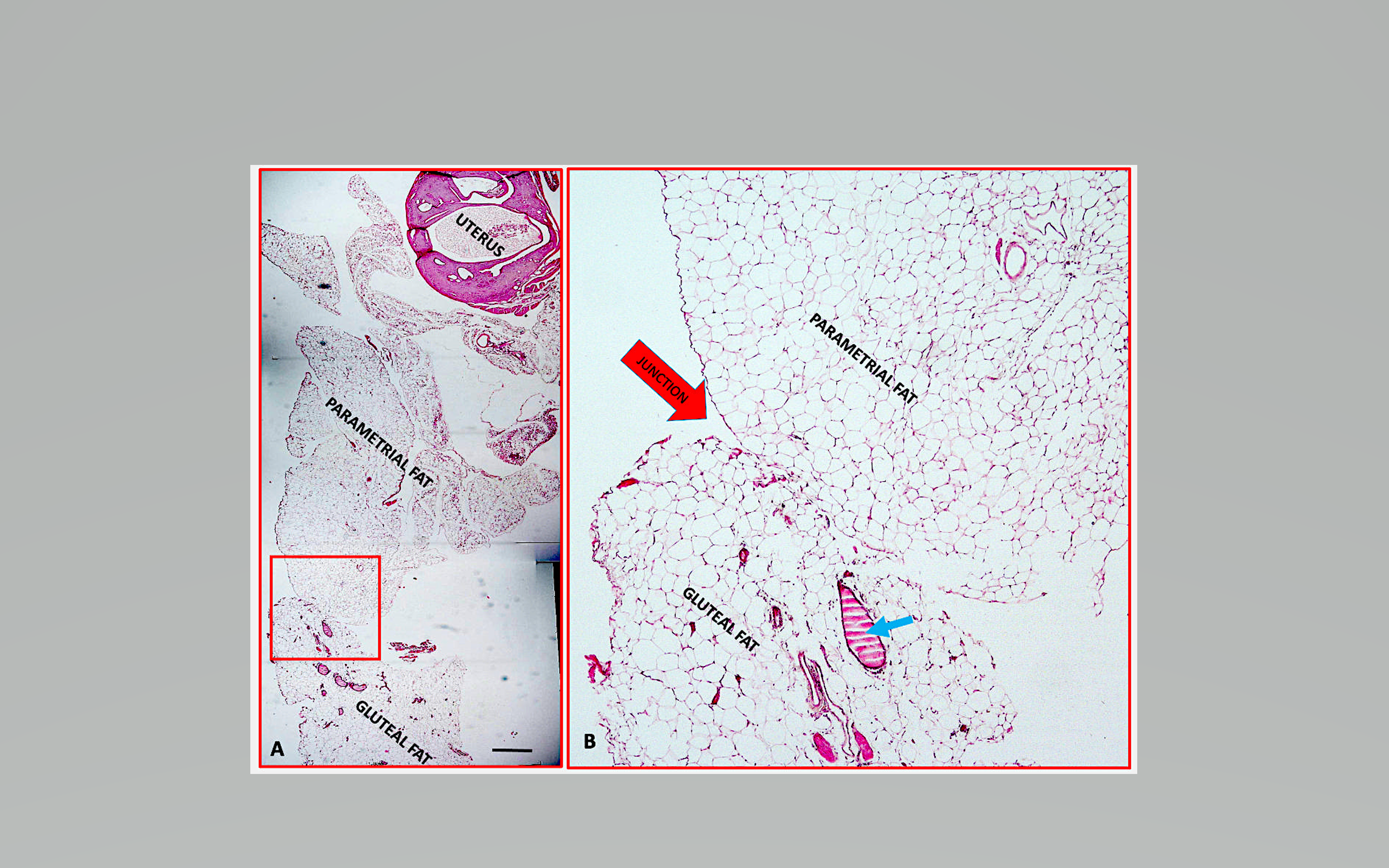

Supplement: Supplementary file 1 [file biomedicines-10-02275-s001.zip › Figure S6.tif]

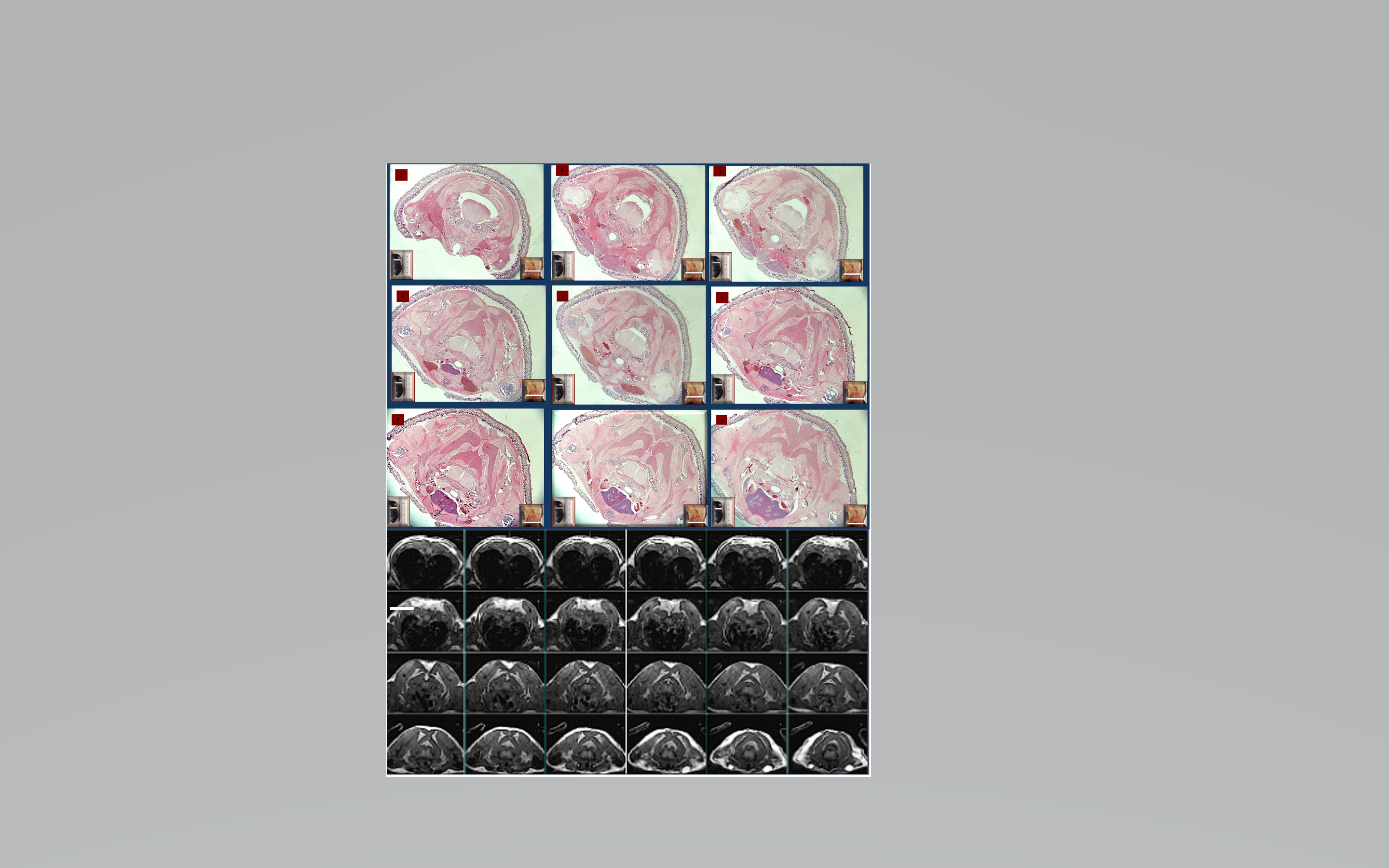

Supplement: Supplementary file 1 [file biomedicines-10-02275-s001.zip › Figure S7.tif]

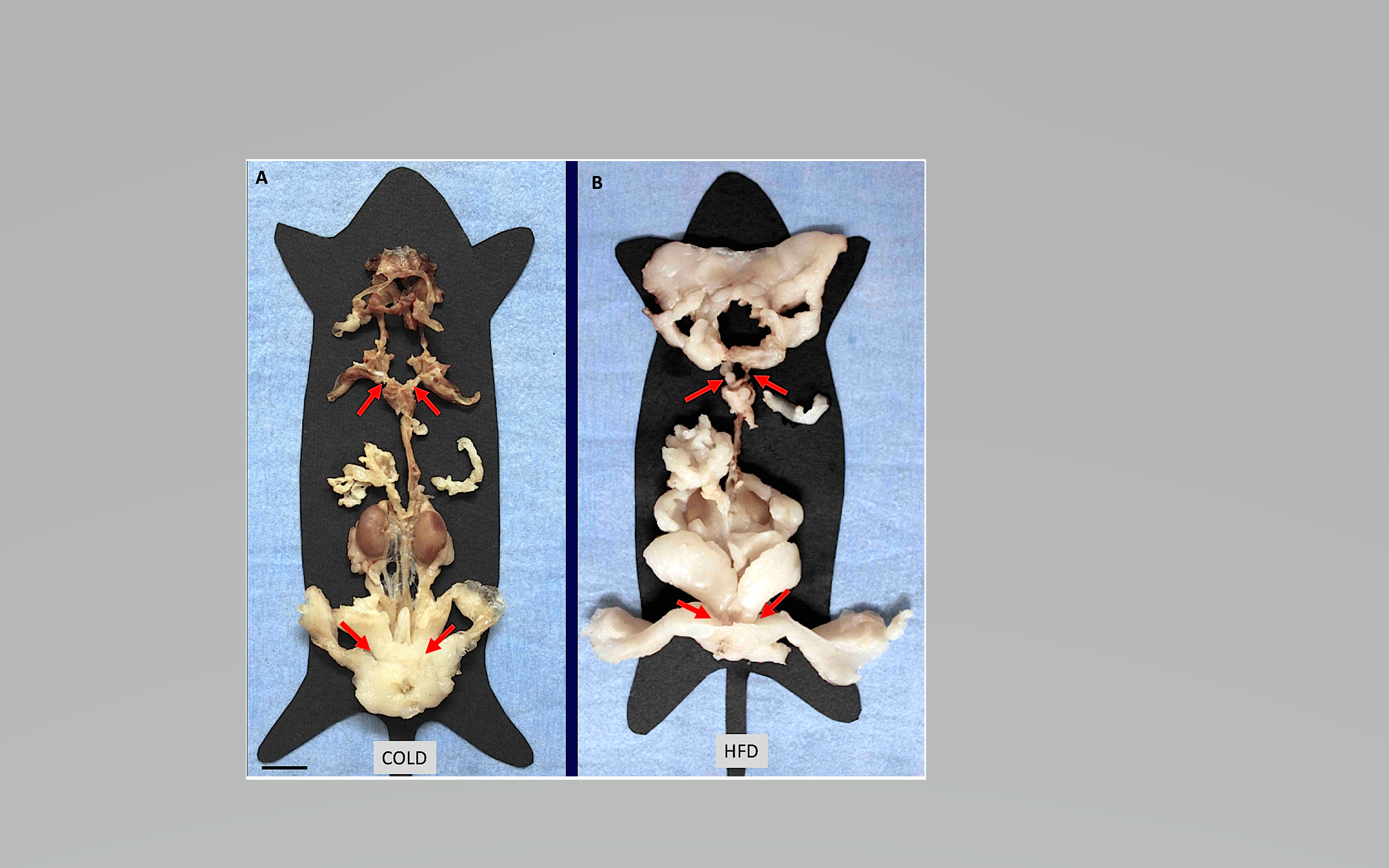

Supplement: Supplementary file 1 [file biomedicines-10-02275-s001.zip › Figure S8.tif]

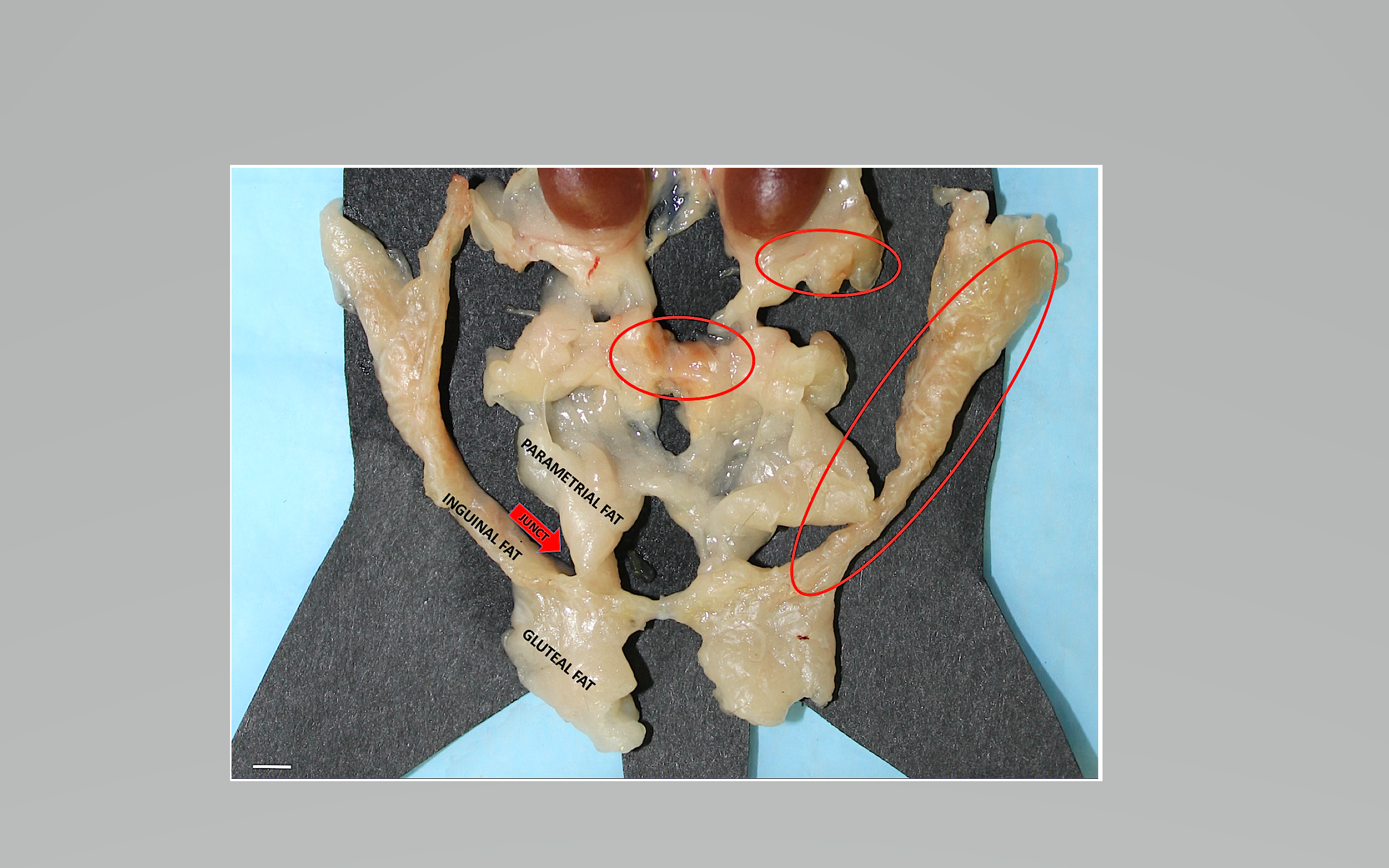

Supplement: Supplementary file 1 [file biomedicines-10-02275-s001.zip › Figure S9.tif]
